# Investigation of early molecular alterations in tauopathy with generative adversarial networks

**DOI:** 10.1038/s41598-023-28081-6

**Published:** 2023-01-13

**Authors:** Hyerin Kim, Yongjin Kim, Chung-Yeol Lee, Do-Geun Kim, Mookyung Cheon

**Affiliations:** Dementia Research Group, Korea Brain Research Institute (KBRI), Daegu, 41062 Republic of Korea

**Keywords:** Computational neuroscience, Diseases of the nervous system

## Abstract

The recent advances in deep learning-based approaches hold great promise for unravelling biological mechanisms, discovering biomarkers, and predicting gene function. Here, we deployed a deep generative model for simulating the molecular progression of tauopathy and dissecting its early features. We applied generative adversarial networks (GANs) for bulk RNA-seq analysis in a mouse model of tauopathy (TPR50-P301S). The union set of differentially expressed genes from four comparisons (two phenotypes with two time points) was used as input training data. We devised four-way transition curves for a virtual simulation of disease progression, clustered and grouped the curves by patterns, and identified eight distinct pattern groups showing different biological features from Gene Ontology enrichment analyses. Genes that were upregulated in early tauopathy were associated with vasculature development, and these changes preceded immune responses. We confirmed significant disease-associated differences in the public human data for the genes of the different pattern groups. Validation with weighted gene co-expression network analysis suggested that our GAN-based approach can be used to detect distinct patterns of early molecular changes during disease progression, which may be extremely difficult in in vivo experiments. The generative model is a valid systematic approach for exploring the sequential cascades of mechanisms and targeting early molecular events related to dementia.

## Introduction

Tau pathology is one of the major attributes of certain neurodegenerative disorders, including Alzheimer’s disease (AD), frontotemporal dementia (FTD), and progressive supranuclear palsy (PSP). In particular, AD, the prototypical tauopathy, is characterised by extracellular amyloid plaques and intraneuronal neurofibrillary tangles, composed of misfolded/aggregated amyloid-beta peptide (Aβ) and tau, respectively^1,2^. The accumulation and aggregation of tau in the brain correlate with synaptic and neuronal loss, resulting in cognitive decline that is also associated with aggregate-mediated cellular interactions and ensuing multifactorial downstream mechanisms^3–6^. Hence, understanding the effects exerted by tau aggregates is of key importance to identify upstream disease-causing events. Recently, neuroimaging data from individuals with mild cognitive impairment, early AD, and AD in general revealed cerebral blood flow reduction and blood‒brain barrier (BBB) breakdown^7^. In vivo studies in mice of different ages have demonstrated the involvement of tau overexpression in a variety of vascular abnormalities, indicating the impact of tau accumulation and toxicity on the neurovascular unit in the early stages and progression of disease^8^. While the effects of vascular Aβ accumulation are better documented, the study of the impact of tau on neurovascular pathways in AD is still in its infancy.

Several transgenic mouse models of tauopathy have been developed to simulate AD-like neuropathology and functional deficits. For example, Tg4510-P301L and 3xTg mice exhibit altered inflammatory responses, blood vessel abnormalities and mitochondrial dysfunctions caused by aggregates of hyperphosphorylated tau^8,9^. One of the most robust models is TPR50, expressing the longest form (2N4R) of tau with a P301S mutation; this model has been shown to have a severe tauopathy phenotype with much higher human tau expression than PS19 mice, a widely used tauopathy model expressing 1N4R tau with a P301S mutation^10^. TPR50 mice were demonstrated to have progressive neuronal and behavioural deficits characteristic of human tauopathies, as they showed cognitive dysfunction at 5 months, abnormally increased microtubule (MT)-related proteins and impaired axonal transport at 5 months or even earlier^11^. Although these models have significantly advanced our understanding of processes underlying neurodegeneration and the development of therapeutic approaches, the detrimental mechanisms of tau aggregation leading to blood vessel damage and the associated neurovascular deregulation are not fully understood.

Deep learning-based approaches are a promising resource for uncovering biological mechanisms, identifying biomarkers, and predicting gene function^12,13^. In addition to numerous biological or clinical imaging-related studies, there have been diverse deep learning-based analyses of genomic or transcriptomic NGS data covering a wide range of topics such as splicing, single-cell transcriptomics, and target genes for therapeutics^14–19^. Developments in the field of neuropathology have included deep learning-based image classification and object detection methods that enable the detection, quantification and classification of plaques or tangles in AD^20–22^. A multi-task deep learning framework was introduced to analyse heterogeneous bulk RNA-seq data from multiple sources of AD postmortem brain tissues^23^. Most of these studies followed supervised learning methods for classification and prediction. Ghahramani et al.’s single-cell transcriptomic study of epidermal cells was the first application of generative adversarial networks (GANs) to omics analysis^24^. Recently, a multi-omics data integration approach for The Cancer Genome Atlas was developed based on GANs^25^. Following a similar network architecture to Ghahramani’s work, we developed a method to utilise bulk RNA-seq data, from which we could produce highly realistic simulated, or “fake”, data suitable for exploring disease progression. We applied our developed GANs for bulk RNA-seq analysis in mice with Aβ aggregation and found that cholesterol biosynthesis was induced by enhanced Aβ production^26^. The gene transition curves (TCs) extracted from the trained generative model identified dynamic perturbations that could be observed with disease progression. In a previous 5xFAD mouse study with three age groups (2, 4 and 7 months), we used only one comparative set of differentially expressed genes (DEGs) to analyse simulated disease progression^26^. However, with only two age groups, such as 3 and 6 months, the investigation of gene perturbations following the previous pipeline might not be adequate for identifying the qualitative features of early phenotype changes.

In this study, to explore all aspects of molecular changes during disease progression, the union set of DEGs for all four comparative pairs (two phenotypes with two time points) was used as input, including early phenotype changes and maturation processes, resulting in four-way TCs. This improvement solved the issues of monotonous and indistinguishable pattern classification of gene expression changes in one-way transition. We hypothesised that the generative model could be used to dissect the early features of tauopathy based on RNA-seq data from mutated human tau transgenic (TPR50-P301S) model mice.

## Results

### GAN model for simulation of tauopathy progression

**Figure 1:**
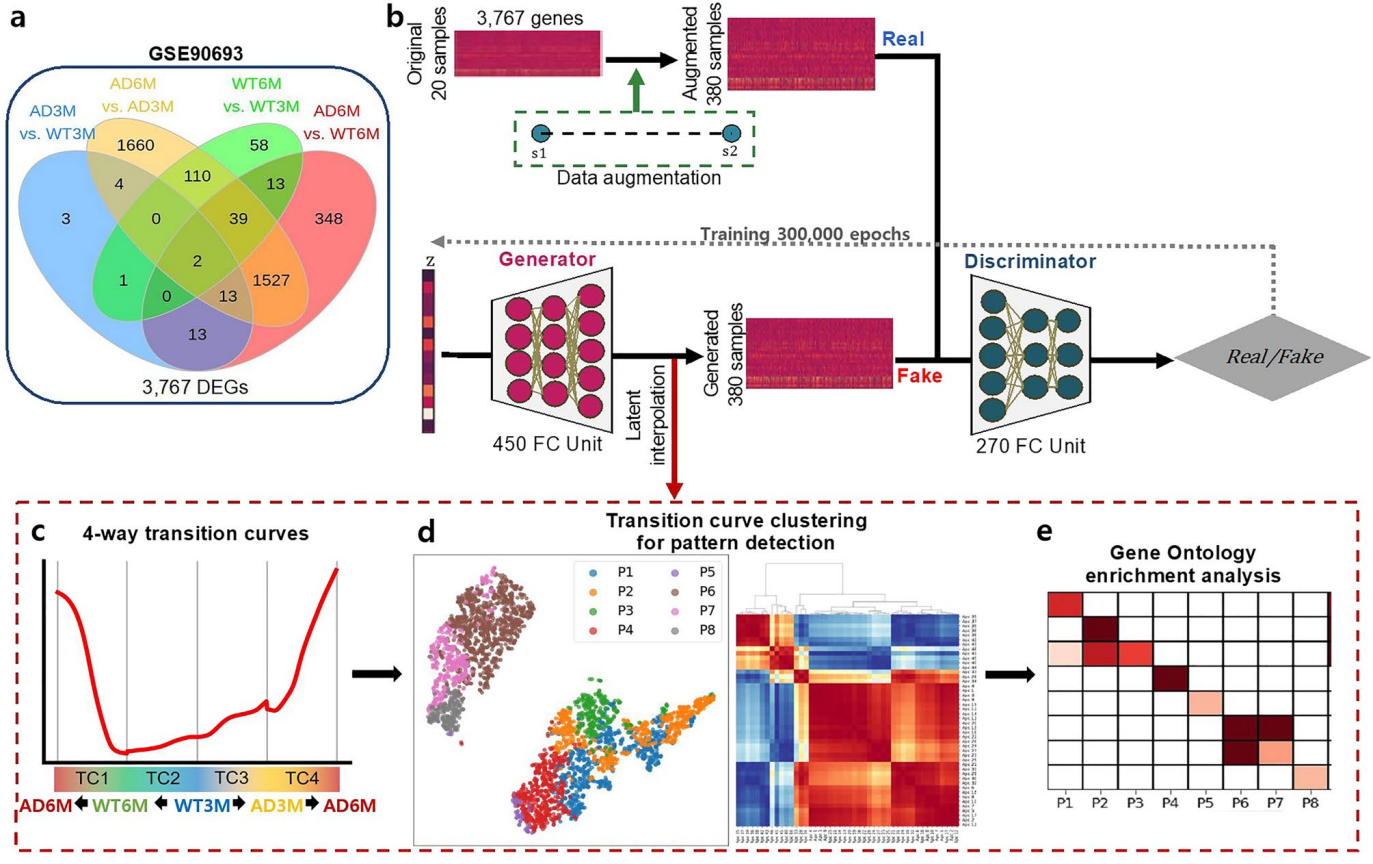
Schematic overview of GAN training and workflow of downstream analyses. (**a**) Venn diagram of DEGs in four comparative pairs from bulk RNA-seq data (GSE90693). (**b**) The generative adversarial network consists of two neural networks simultaneously training and competing against each other (see Supplementary Fig. 2). (**c**) Representation of four-way TCs: TC1 (late phenotype changes, WT6M → AD6M), TC2 (WT maturation, WT3M → WT6M), TC3 (early phenotype changes, WT3M → AD3M), and TC4 (AD maturation, AD3M → AD6M). (**d**) Four-way TCs are clustered and grouped according to pattern similarities. (**e**) Gene Ontology enrichment analysis by TC pattern. AD, Alzheimer’s disease; DEGs, differentially expressed genes; FC, fully connected layer; GOBP, Gene Ontology biological process; M, months; P, pattern group; TC, transition curve; WT, wild type.

From a publicly available RNA-seq dataset derived from TPR50 Tau P301S transgenic mice (GSE90693)^10^, we performed analyses with two time points (3 and 6 months, labelled 3 M and 6 M, respectively) and two phenotypes (wild-type mice, referred to as WT, and TPR50-P301S mice, referred to as AD). From the original data, we selected 20 C57BL6-strain mouse cortex samples. After reprocessing the RNA-seq data, we obtained a union set of 3767 DEGs from four comparative pairs: WT maturation (WT6M vs. WT3M), AD maturation (AD6M vs. AD3M), early phenotype changes (AD3M vs WT3M) and late phenotype changes (AD6M vs. WT6M) (Fig. 1a, Supplementary Fig. 1 and Gene Ontology enrichment analysis results in Supplementary Table 1).

A schematic overview of GAN training and downstream analyses is shown in Fig. 1b–e. Using pairwise linear interpolation, the data of 20 samples were augmented to 380 samples in each group, which were used as real data. The evaluation of the generated gene expression profiles showed that the generated profiles were closely correlated with the real data, and one group of samples formed a single cluster in the t-SNE plot (Supplementary Fig. 3).

### Identification of four-way TCs and clustering

**Figure 2:**
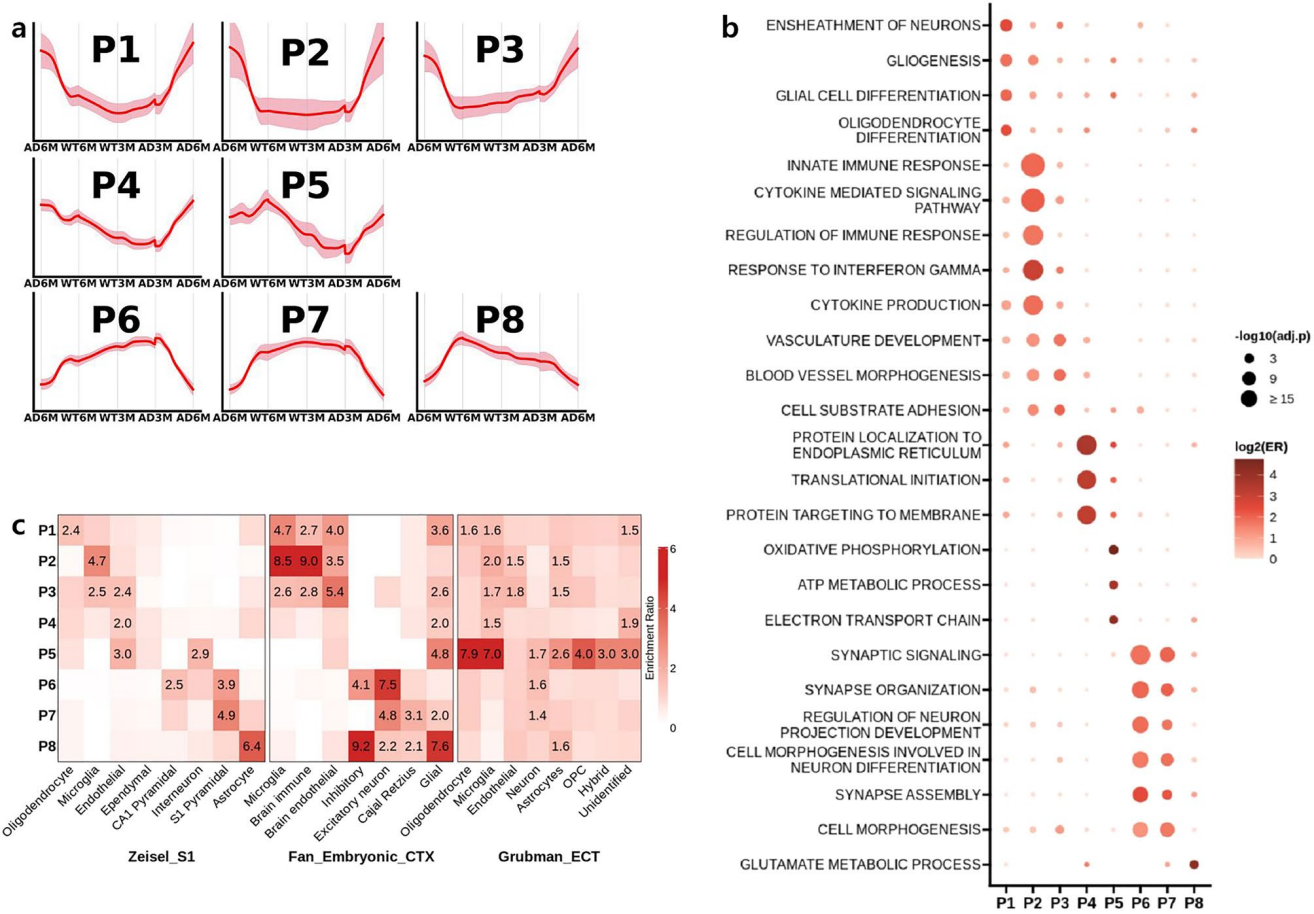
The eight pattern groups of GAN TCs. (**a**) Each pattern group showed up- or downregulation in the four-way TCs. The red line represents the average TCs in each pattern group. (**b**) Enriched Gene Ontology biological process terms of each pattern group. Statistical significance (adj.p, dot size) and ER (colour scale). (**c**) Cell-type enrichment of each pattern with mouse primary somatosensory cortex (Zeisel_S1), human embryonic cortex (Fan_Embryonic_CTX), and entorhinal cortex from postmortem AD patients (Grubman_ECT). adj.p, adjusted p value; ER, enrichment ratio; GAN, generative adversarial network.

After training the model, we extracted the generated gene expression profiles from the latent space interpolation and obtained four-way TCs of 3767 genes. Here, the four-way TCs were defined as follows: TC1 (late phenotype changes, WT6M → AD6M), TC2 (WT maturation, WT3M → WT6M), TC3 (early phenotype changes, WT3M → AD3M), and TC4 (AD maturation, AD3M → AD6M) (Fig. 1c). According to the technical definition, TCs are smooth and nonlinear interpolated changes between two states to capture intermediate features manipulated by vector arithmetic in the latent space. The 3767 TCs were merged into 56 clusters using affinity propagation clustering (APC, Supplementary Fig. 4). Although some clusters appeared to be highly similar, they were separate due to the distinct scales of expression levels (y-axis). Among 56 clusters, we grouped the 46 clusters of 3381 TCs into eight pattern groups (P1 to P8) by direction (upwards or downwards) for each TC (Fig. 2a and Supplementary Table 2 for lists of genes for eight pattern groups). The remaining 10 clusters of 388 TCs could not be merged because they presented undefined patterns. The description of TCs and numbers of genes in each group are summarised in Table 1.

**Table 1:** Four-way TCs and number of genes in each pattern group.

	TC1 Late phenotype (AD6M ← WT6M)	TC2 WT maturation (WT6M ← WT3M)	TC3 Early phenotype (WT3M → AD3M)	TC4 AD maturation (AD3M → AD6M)	Number of genes
P1	▲	▲	△	▲	643
P2	▲	–	–	▲	567
P3	▲	▽	▲	▲	240
P4	△	▲	▽	▲	477
P5	▼	▲	▼	▲	48
P6	▼	▼	▲	▼	871
P7	▼	▽	▽	▼	335
P8	▼	▲	▼	▼	200

▲: up; ▼: down; △: even/up; ▽: even/down; –: even; AD, Alzheimer’s disease; M, months; P, pattern group; TC, transition curve; WT, wild type.

### Upregulation of gliosis and immune responses in late phenotype changes (P1 and P2)

**Figure 3:**
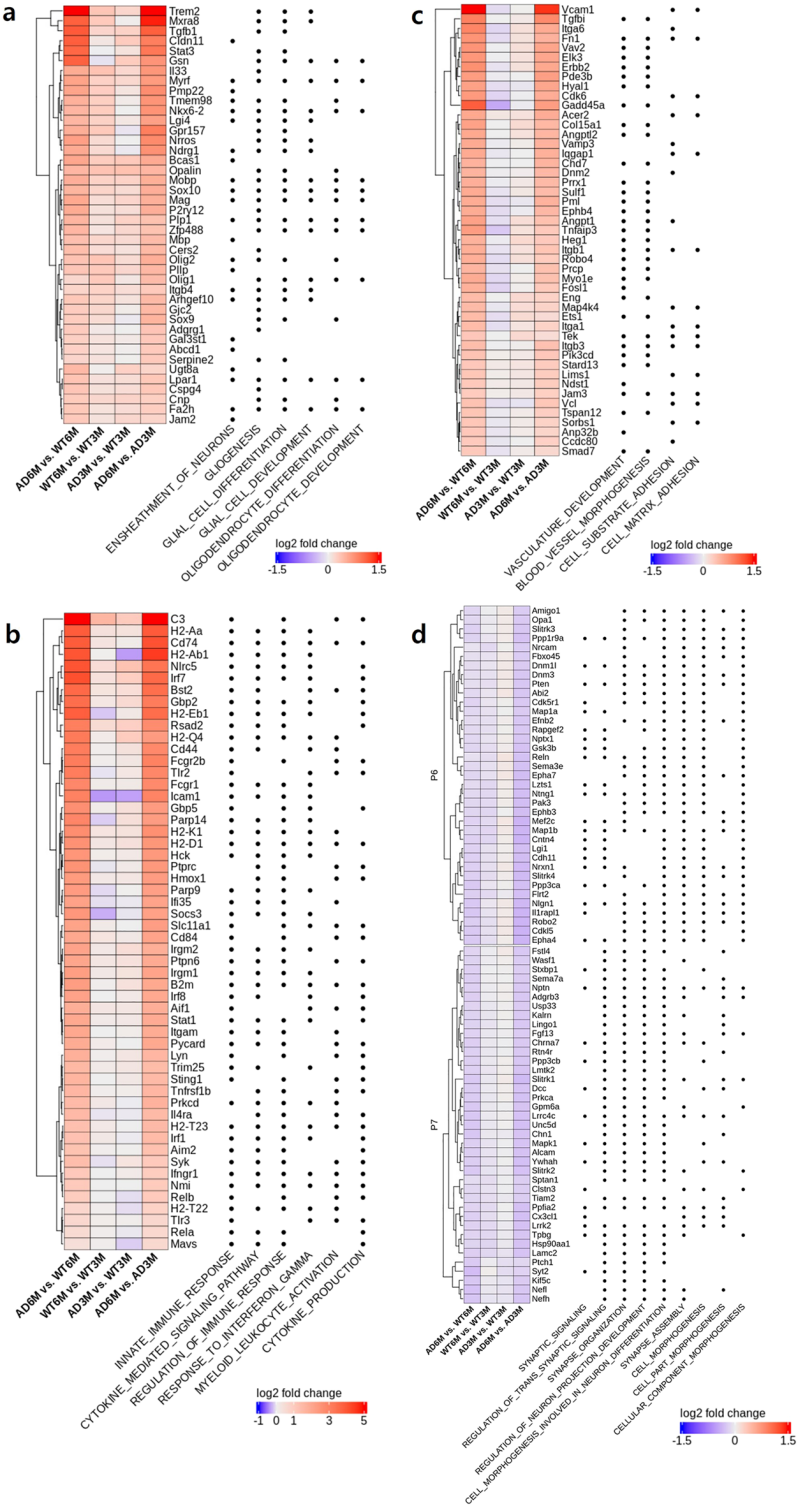
Recurrently annotated genes were selected and are shown with the associated Gene Ontology biological process terms. The numbers of genes were as follows: (**a**) 42 in P1, (**b**) 54 in P2, (**c**) 47 in P3, (**d**) 37 in P6, and 39 in P7.

Three of the pattern groups, P1, P2, and P3, showed strong upregulation in late phenotype changes (Table 1 and Fig. 2a). Group P1, with 10 clusters and 643 genes, was characterised by upwards patterns in most TCs. The dominant GOBP annotations for this group were ensheathment of neurons (enrichment ratio [ER] = 5.57, adjusted *p* value [*adj.p*] = 6.72E−8), gliogenesis (ER = 3.58, *adj.p* = 2.38E−7), and glial cell differentiation (ER = 3.90, and *adj.p* = 9.76E−7), supporting the role of altered glial function as an underlying dynamic feature of neurological disorders (Fig. 2b)^27^. The dominant corresponding cell types were oligodendrocytes and glial cells, which activate the immune response in the central nervous system (Fig. 2c). Figure 3a depicts the expression of 42 genes in P1 that were recurrently annotated in notable GOBP terms. The expression pattern of these genes reflects the direction of the P1 pattern (i.e., upregulation in late phenotype changes and WT/AD maturation; slight upregulation in early phenotype changes). Notably, *apolipoprotein E* (*Apoe*), *TYRO protein tyrosine kinase binding protein* (*Tyrobp*), and *triggering receptor expressed in myeloid cells 2* (*Trem2*), which are known high-risk genes for the development of late-onset AD and are highly expressed in microglia^28^, showed strong upregulation in both late phenotype changes and AD maturation.

The P2 group, with 11 clusters and 567 genes, showed upregulation of gene expression in late phenotype changes and AD maturation but not in WT maturation or early phenotype changes, reflecting the general pathological phenotype in late stages of neurodegeneration (Table 1). The dominant GOBP annotations comprised inflammatory and immune responses, such as innate immune response (ER = 3.93, *adj.p* = 1.57E−37), cytokine-mediated signalling (ER = 4.37, *adj.p* = 2.33E−36), and response to interferon gamma (ER = 7.78, *adj.p* = 5.25E−26) (Fig. 2b). Neuroinflammation has been reported as an important component of AD pathology, and many experimental, genetic, and epidemiological studies have shown extensive upregulation of genes associated with the immune system in this disease^29,30^. Many genes that play a role in the innate immune system in the brain were included in the P2 group, such as CD antigens (*Cd14*, *Cd44*, *Cd74*, and *Cd84*), interferon-induced genes (*Ifit1*, *Ifit2*, *Ifit3*, and *Ifitm3*), and Toll-like receptor genes (*Tlr2*, *Tlr3*, *Tlr7*, *Tlr9*, and *Tlr13*)^31^. Accordingly, P2 genes were predominantly associated with microglia, the cellular mediators of neuroinflammation (Fig. 2c). The 54 genes recurrently annotated in immune-related GOBP terms were strongly upregulated in late phenotype changes and AD maturation and slightly downregulated or unchanged in early phenotype changes and WT maturation (Fig. 3b). Several genes in P2 were explicitly associated with cascade regulation of immune responses, including histocompatibility 2 genes (*H2-Aa*, *H2-Ab1*, *H2-Eb1*, *H2-Q4*, *H2-K1*, *K2-D1*, *H2-T23*, and *H2-T22*) for antigen presentation, signal transducer and activator of transcription genes (*Stat1*, *Stat3*, and *Stat6*) and proteins related to nuclear factor kappa-B signalling (*Nfkbia*, *Nfkbiz*, *Rela*, and *Relb*) for cytokine production. In particular, *complement component 3* (*C3*), the strongest upregulated gene in both late phenotype changes and AD maturation, is a major component of the complement cascade, and deficiency of C3 mitigates neurodegeneration and neuronal loss in the P301S tauopathy mouse model^32^.

### Upregulation of vasculature development-associated genes in both early and late phenotype changes (P3)

The P3 group, with 6 clusters and 240 genes, indicated an involvement in early and late pathological processes but was not associated with WT maturation (Table 1). Observing the stronger upregulation in P3 than in P1 at early phenotypic changes, we mainly focused on P3 to dissect triggering features in early tauopathy. The primary GOBP annotations were as follows: vasculature development (ER = 3.47, *adj.p* = 3.56E−7), blood vessel morphogenesis (ER = 3.69, *adj.p* = 3.56E−7), and cell substrate adhesion (ER = 4.15, *adj.p* = 9.78E−5) (Fig. 2b); correspondingly, P3-associated genes were enriched in brain endothelial cells (Fig. 2c). The 47 genes recurrently annotated in the significant pathways were upregulated in early/late phenotype changes and AD maturation and slightly downregulated in WT maturation, including integrin genes (*Itga1*, *Itga6*, and *Itgb1*), *fibronectin 1* (*Fn1*), *angiopoietin 1* (*Angpt1*), and *angiopoietin-1 receptor* (*Tek*) (Fig. 3c). Among the early-upregulated genes, *angiopoietin 1* (*Angpt1*) primarily acts on the regulation of angiogenesis and is implicated in nervous system development.

**Figure 4:**
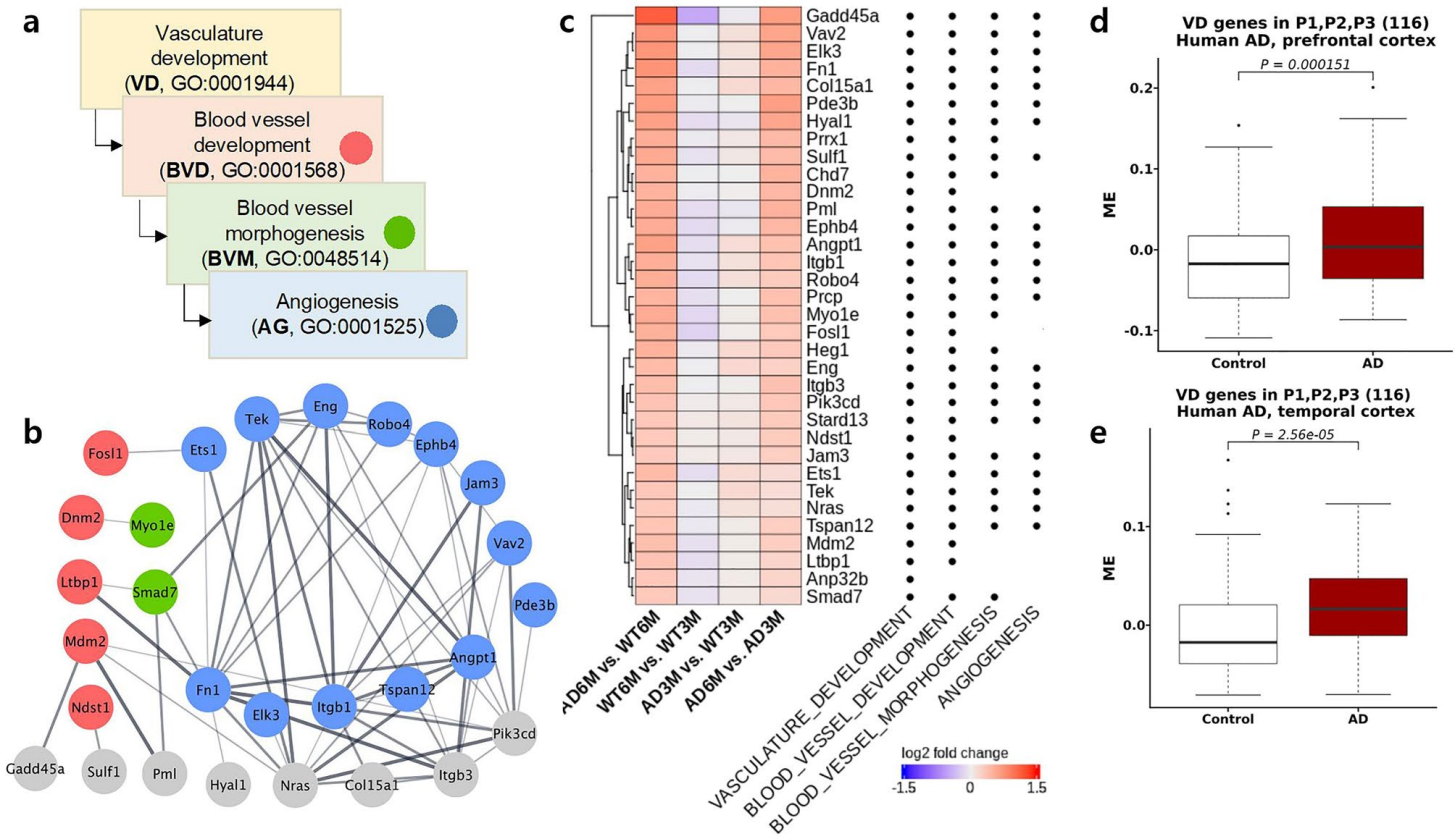
In-depth analysis of angiogenesis. (**a**) Gene Ontology tree of angiogenesis-related processes. (**b**) Protein‒protein interactions among the 20 VD genes with associated GO pathways (according to the colour code in (**a**)). (**c**) Gene expression heatmap for log2 fold changes of 34 VD genes in four comparative pairs. (**d**,**e**) Module eigengenes (MEs) for 116 VD genes in P1, P2, and P3 in the human AD prefrontal cortex (ROSMAP) and temporal cortex (Mayo Clinic). VD, vasculature development; ME, module eigengene.

Angiogenesis is a complex process consisting of several discrete steps beginning with endothelial activation. To comprehensively analyse the P3 gene expression profile regarding the regulation of angiogenesis (GO:0001525), we examined upper-level angiogenesis pathways using the Mouse Genome Informatics database (http://www.informatics.jax.org/)^33^. Of the 240 genes in P3, 23 were associated with angiogenesis, while 28 genes (including the angiogenesis-related genes) were associated with blood vessel morphogenesis (GO:0048514). A further 33 genes were related to blood vessel development (GO:0001568), and 34 genes were related to vasculature development (GO:0001944) (Fig. 4a). The protein‒protein interactions for 20 out of 34 genes associated with vasculature development showed a tight connection between *Angpt1* and *Tek*, reflecting the role of *Angpt1* as the major agonist for *Tek* (Fig. 4b). Aside from *Angpt1*, most of the genes associated with vasculature development that were identified in P3 showed slight upregulation in early phenotype changes, suggesting that vascular changes occur at early stages of tau pathology (Fig. 4c).

While genes in all P1, P2, and P3 represent vasculature development, this association was stronger in P2 (*adj.p* = 1.17E−08) than in P3 (*adj.p* = 4.13E−07) and P1 (*adj.p* = 3.77E−02) (Fig. 2b). The number of P3 genes, 34, was relatively small compared to that of P1, 43, (Supplementary Fig. 5a) and P2, 64, (Supplementary Fig. 5b), which implies that a small portion of vasculature development-associated genes were upregulated in early phenotype changes, while a larger portion of those genes were upregulated at a later stage, concomitant with the initiation of immune responses. In addition, employing recently identified markers specific to brain vascular cells^34^, we observed that genes in P2 were significantly enriched in arteries (*adj.p* = 1.11E−05) and veins (*adj.p* = 0.0037), while genes in P3 were enriched in capillaries (*adj.p* = 0.03). Notably, P1, P2, and P3 failed to display any changes in WT maturation, corroborating the idea that the vascular changes in capillaries at the early stage are associated with pure tauopathy.

There are several human studies that provide strong evidence for an association between tauopathy and vascular dysregulation^8^. We performed additional analyses with two independent cortex RNA-seq datasets from postmortem patients with AD to validate the relevance of the observed changes (Supplementary Fig. 6). The 116 vasculature development–associated genes in P1, P2, and P3 were highly expressed in both the prefrontal (*p* = 1.12e−04) and temporal (*p* = 2.56e−05) cortices of AD patients (Fig. 4d,e).

On the basis of this combined evidence, we propose that vasculature development and gliosis processes trigger the immune responses observed in early tauopathy.

### Metabolomic and mitochondrial dysregulation in early phenotypic changes (P4 and P5)

The P4 group, with five clusters and 477 genes, displayed an upregulation in the WT and AD maturation processes, while slight up- and downregulation was observed in late and early phenotype changes, respectively (Table 1 and Fig. 2a). The dominant pathway annotations of the P4 group were as follows: cotranslational protein targeting to membrane (ER = 16.74, *adj.p* = 2.34E−31), protein localisation to endoplasmic reticulum (ER = 11.89, *adj.p* = 6.45E−26), and translational initiation (ER = 9.07, *adj.p* = 3.10E−22) (Fig. 2b and Supplementary Table 3). Multiple genes coding for ribosomal proteins (RPs), including *Rps9*, *Rps11*, and *Rps16*, were found in this group. The interaction of tau with dozens of canonical RNA-binding proteins and RPs has been reported, suggesting its participation in RNA granule metabolism^35^. That tau is involved in protein synthesis has further been demonstrated through the dysregulation of ribosomal proteins in tau and Aβ mouse models^36–38^. Since RPs ubiquitously regulate multiple cellular processes, we could not find any significantly enriched brain cell types (Fig. 2c and Supplementary Table 3).

The smallest group, P5, contained two clusters and 48 genes and displayed a unique pattern that indicated upregulation in both maturation processes and downregulation in both phenotype changes (Table 1 and Fig. 2a). The dominant GOBP annotations were mitochondrial metabolic processes, such as oxidative phosphorylation (ER = 27.20, *adj.p* = 5.47E−04), ATP synthesis-coupled electron transport (ER = 32.53, *adj.p* = 1.92E−03), and ATP metabolic processes (ER = 12.79, *adj.p* = 4.0E−3) (Fig. 2b and Supplementary Table 3); consequently, most genes in these pathways were mitochondria-related genes, including *mt-Nd4l*, *mt-Co2*, and *mt-Nd3*, and P5 genes were highly enriched in most cell types of human AD specimens. (Fig. 2C and Supplementary Table 3). Hence, mitochondrial metabolic processes were downregulated in both early and late tauopathy and upregulated in WT maturation.

Overall, we observed that tau impaired metabolomic and mitochondrial processes in early tauopathy, which was reflected by our GAN-based simulation that produced these two distinct patterns of molecular changes, P4 and P5.

### Downregulation of synaptic signalling in late phenotype changes (P6, P7, and P8)

The pattern of the largest group, P6, with five clusters and 871 genes, suggested a slight increase in early phenotype changes, similar to P1 and P3, but a decrease in late phenotype changes, AD maturation, and WT maturation (Table 1 and Fig. 2a). The GOBP annotations were as follows: synaptic signalling (ER = 3.53, *adj.p* = 3.09E−24), regulation of trans-synaptic signalling (ER = 4.19, *adj.p* = 6.04E−20), and synapse organisation (ER = 3.95, *adj.p* = 1.05E−17) (Fig. 2b and Supplementary Table 3). The dominant brain cell type was neurons (Fig. 2c and Supplementary Table 3). Interestingly, several P6 genes involved in neuronal differentiation and synapse assembly were slightly upregulated in early phenotype changes (Fig. 3d), which is consistent with evidence suggesting that the early stages of tauopathy are associated with alterations in both pre- and post-synaptic turnover rates in the rTg4510 mouse model^39^.

The P7 group, containing four clusters and 335 genes, was characterised by a pattern similar to that found in P6 (Table 1 and Fig. 2a). Although these groups showed different patterns in early phenotype changes and the number of genes in P6 was 2.5-fold higher than that in P7, the majority of enriched GOBP terms of these groups overlapped (Fig. 2b). The enriched GO terms of P7 were synaptic signalling (ER = 4.08, *adj.p* = 7.39E−13), cell morphogenesis (ER = 3.29, *adj.p* = 1.21E−11), and cell part morphogenesis (ER = 3.75, *adj.p* = 2.03E−10) (Supplementary Table 3). Interestingly, the significantly enriched brain cell type was the Cajal–Retzius cell, which plays a crucial role in cellular development and synaptogenesis (Fig. 2c and Supplementary Table 3). Collectively, the downregulation of genes in Group P7 impaired synaptogenesis, while the upregulation of genes in P6 contributed to enhanced synaptic plasticity and stability in early tauopathy (Fig. 3d).

Finally, P8 comprised three clusters and 200 genes, which followed a pattern that was opposite to that observed in P6 and P7 regarding WT maturation (Table 1 and Fig. 2a). The enriched GO pathways were related to glutamate processes, and the dominant cell type was the astrocyte, which implies that the genes within this group may be related to functions of neuronal projection by astrocytes (Fig. 2b,c and Supplementary Table 3). It has been reported that the expression of proteins related to glutamate homeostasis is significantly altered in the superficial cerebral cortex of 3- and 5-month-old P301S tau mice^40^.

### Validation of TC patterns using WGCNA modules

**Figure 5:**
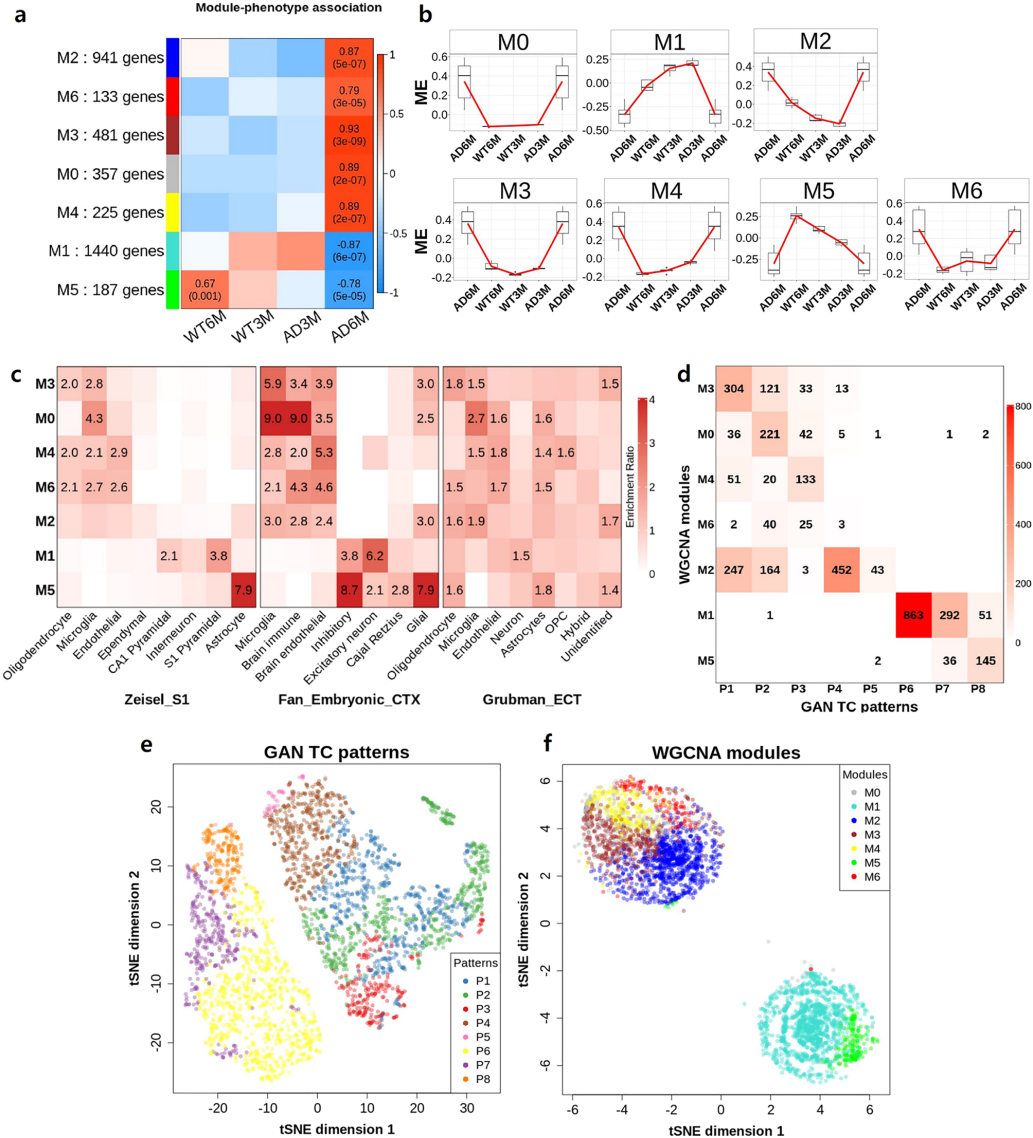
Validation of the simulated TC patterns using WGCNA. (**a**) Using WGCNA, we detected seven modules. The numbers denoted in the heatmap show correlations and the corresponding p values for associations of module patterns with phenotypes and ages. (**b**) Associations between patterns and eigengenes in modules. (**c**) Cell-type enrichment heatmap for each module. (**d**) Number of overlapping genes between GAN TC patterns and WGCNA module patterns. The results of t-SNE plots for (**e**) GAN TC patterns and (**f**) WGCNA modules. Each label in the plot was coloured by its GAN TC patterns and WGCNA modules. TC, transition curve; WGCNA, weighted gene co-expression network analysis.

To validate the simulated TC patterns of our GAN model, we performed weighted gene co-expression network analysis (WGCNA); this methodology has been widely used for co-expression module detection and analysis with phenotype-associated changes in AD studies^10,28,41^. The WGCNA with 3767 DEGs yielded seven significant modules (M0–M6); each of these contained between 133 and 1440 genes (Fig. 5a and Supplementary Fig. 8). Each module displayed a different eigenvalue pattern (Fig. 5b). We compared the WGCNA results with the GAN TC patterns and found that P1, P2, P4, and P6 were highly similar to M3, M0, M2, and M1, respectively (Figs. 2a and 5b). The enriched cell types were found to be highly conserved between P1 and M3 (glial cells), between P2 and M0 (microglia), between P3 and M4 (endothelial cells), and between P6 and M1 (neurons) (Figs. 2c and 5c). We next analysed the preservation of the genes between TC pattern groups and WGCNA modules; Fig. 5d shows the numbers of common genes in the corresponding GAN TC patterns and modules. As expected, the pairs P6-M1, P4-M2, and P1-M3 showed overlaps containing many genes, indicating conservation. Interestingly, M1 contained the genes that were also found in both P6 and P7, while there was no correlation between any TC pattern group and M6. In addition, t-SNE was applied to reduce the dimensionality of GAN TC patterns and WGCNA modules (Fig. 5e,f). The two t-SNE plots showed two distinct clusters in regard to functional and cell-type specificity. The t-SNE of GAN TC patterns showed that P1, P2 and P3 (i.e., gliosis and immune response in glial cells) were closely correlated as well as P6 and P7 (i.e., synapse dysfunction in neurons). Similarly, the t-SNE of WGCNA modules revealed two large clusters: the larger of the two represents the very closed mixture of M0, M2, M3, M4 and M6, and the other cluster contains neuron-specific modules, M1 and M5. Gene Ontology enrichment analysis for each module confirmed common GO terms between TC pattern groups and WGCNA modules (Supplementary Fig. 8c and Supplementary Table 4). However, the enrichment of genes associated with gliosis and vasculature development observed in P1 and P3 appeared to be weak in M3 and M4, which may be due to the strong immune responses. Overall, whereas the WGCNA approach is specialised to detect the gene modules with expression profiles of discrete time points, the GAN-based approach is suitable to examine patterns of molecular changes in a continuous manner based on latent space interpolation.

### Inspection for sample populations of four groups having a small sample size

Developing a deep learning-based analytic method to utilise bulk RNA-seq data having a small sample size is a great strength of this work, but it also contains one concern that needs to be checked. We used four age- and phenotype-dependent groups in this study. Samples in each group must be clearly different from samples in other groups because the latent interpolation of GANs depends on fluctuation of gene expression levels. That is, it must be proved that the features of the groups are well separated from each other and that the populations of each group are clearly different. For this purpose, we performed permutation test analysis by randomise samples and checked the results of WGCNA and GANs analyses (Methods and Supplementary Figs. 9–13). By performing 20 permutation trials, we observed loss of significance of WGCNA modules, randomised trends of TCs, and well separated distributions of original data. We confirmed that the four populations of four groups have clear distinctive distributions depending on age and phenotype differences.

## Discussion

In this study, we applied a deep learning-based generative model to dissect distinct early molecular features in tauopathy. In our previous study, we utilised GANs and discovered that enhanced cholesterol biosynthesis was associated with the generated expression profiles of disease progression in 5xFAD model mice^26^. These generated nonlinear expression profiles demonstrated that latent space interpolation produces meaningful gene expression predictions based on gene expression interdependencies (TC) and not simply on averaging of gene expression profiles^24^. However, we could not examine the early features of tau pathology using one-way TC owing to the insufficient number of age groups. Here, to overcome this limitation, we trained our generative model with the union set of 3767 DEGs of four comparative pairs and simulated the process of molecular perturbations in four directions, taking into account early and late phenotype changes in addition to WT and AD maturation using latent space interpolation. The 3767 simulated TCs were clustered and grouped according to gene expression pattern similarities, and each pattern group presented distinct GOBP terms and brain-specific cell types. We found that applying GANs enables a more robust approach than conventional analytic methods, such as gene clustering or correlation analysis, for unravelling early and late molecular mechanisms underlying disease progression.

We identified eight groups of TC patterns from 3381 TCs, and each pattern group was characterised and defined in its association with tauopathy. The in-depth analysis of the P1, P2, and P3 groups represented the cascade processes in tauopathy, where tau aggregates alter angiogenesis-related molecular compositions in cerebral blood vessels, leading to the initiation of immune responses. In addition, the P4 and P5 groups were characterised by a downregulation of genes in early phenotype changes, which indicates that tau impairs metabolomic and mitochondrial processes in early tauopathy. Finally, P6, P7, and P8 presented downregulation of genes in late tauopathy specifically in neurons, suggesting that tau aggregates correlate with synaptic dysfunction.

Our findings identified changes in vascular development and gliosis as early features of tauopathy, which precede the initiation of immune responses; this is consistent with recent reports that point to the impact of pathological tau on BBB integrity and functionality^8^. In the AD brain, large populations of endothelial cells are activated by angiopoietins due to brain hypoxia and inflammation^42^. As previously suggested, *Angpt1,* as a potential biomarker, promotes amyloid plaque formation and Aβ generation in APP/PS1 mice; moreover, Ang1 serum levels are elevated in patients with AD as a result of hypoxia-induced angiogenesis^43,44^. BBB breakdown and vascular impairments are suggested as early pathological events, while causality between BBB breakdown and tauopathy remains unclear. It should be noted that vascular changes in proteinopathy could be clarified thorough longitudinal studies and validated as an early upstream mechanistic event.

An additional distinct pattern that we identified in early tauopathy is mitochondrial dysfunction. As a mediator of mitochondrial dysfunction in both in vitro and in vivo models and in human tauopathy^8^, tau aggregates impair mitochondrial localisation, distribution, and dynamics, alter ATP and reactive oxygen species production, and compromise oxidative phosphorylation systems. Although future studies will be required to fully understand the mitochondrial effects of tau, our results support the hypothesis that mitochondrial dysfunction may be an early mechanism in tauopathy.

In this work, we proposed a GAN-based analysis procedure to utilise bulk RNA-seq data; this method has a unique advantage in producing highly realistic fake data for our purposes. However, detailed cell-type specific molecular features could be described roughly compared to studies based on single-cell RNA-seq data. In the case of noisy and sparse data such as scRNA-seq, it is still difficult to make highly similar fake data. It is notable that the correlation coefficients between real and fake data are 0.7–0.9 for the scRNA-seq GANs and 0.9–0.99 for our bulk RNA-seq GANs. Hence, it is worthwhile to develop a GAN-based analysis pipeline for bulk data at present, expecting high-quality single-cell data in the future. In particular, many available AD-related bulk RNA-seq data for postmortem human brain tissues provided by the AD consortium have stimulated us to devise a platform of GAN-based analysis to analyse heterogenous and integrated datasets, which is an ongoing topic of our study.

In summary, we believe that the early features of tauopathy as suggested by our GAN approach could contribute to the identification of potential biomarkers and add to the current understanding of pathological mechanisms. Although the application of GANs to uncover gene expression profiles is highly complex and the achievement of consistent results is difficult, it will be worthwhile to develop and improve this method to detect subtle molecular changes at an early stage, which may be extremely difficult in in vivo experiments. The approach presented here may provide potential guidelines to create a platform for systematically predicting the sequential cascades of mechanisms and targeting early molecular events in relation to dementia, thus facilitating the development of drugs and therapeutic strategies.

## Methods

### Deep learning-based GAN workflow

The original GAN model consists of two adversarial networks, generative and discriminative, whereby the former generates simulated data and the latter evaluates whether the generated data are real or fake^45^. These two networks compete with each other and are trained to improve their performance. After training, we extracted the four-way TCs from the latent interpolation and performed clustering and grouping of TC patterns to distinguish up- and downregulated gene sets. Finally, we defined the grouped patterns based on enriched complex pathological pathways using biological functional Gene Ontology biological process (GOBP) annotations.

### Dataset and preprocessing

For analysis of the TPR50 dataset^10^, Sequence Read Archive files were downloaded from the NCBI Gene Expression Omnibus (GEO) database^46^ with accession GSE90693 (https://www.ncbi.nlm.nih.gov/geo/query/acc.cgi?acc=GSE90693), and raw fastq files were generated using the fastq-dump command from the NCBI SRA toolkit v.2.10.5^47^. Data cleaning and adapter trimming were executed using Trimmometric v.0.38^48^. Alignment to the mouse reference genome GRCm38 (Ensemble release 96) was performed using HISAT2 v.2.1.0^49^, and reads in BAM files were subsequently sorted by coordinate using samtools v.1.7^50^. Genes were quantified using HTSeq-counts v.0.12.4^51^. Gene counts were filtered to remove low read counts (> 80% of samples with > 10 reads) as previously described^10^. Differential gene expression analysis was conducted using the DESeq2 v.1.28.1 package^52^ in R v.4.0.3. We selected *n* = 5 samples for each group (two phenotypes: control [WT] and disease [AD], and two time points: 3 and 6 months, Supplementary Fig. 1a). The gene counts were normalised and fitted to the generalised linear model with a negative binomial distribution. Then, we performed a Wald test to select differentially expressed genes (DEGs) from the normalised data by applying a false-discovery rate threshold of 0.05 using multiple testing with the Benjamini–Hochberg method^53^ and a minimum absolute log2 fold change of 0.3 (see Supplementary Fig. 1b for the number of DEGs with different log2 fold change cut-offs). Supplementary Table 1 details all differentially expressed gene sets satisfying the criteria for all comparisons. Furthermore, we carried out regularized log transformation (RLD) for the selected gene count data, which transformed the skewed distribution to be symmetric, and subsequently used them as the training dataset.

### Gene ontology and cell-type enrichment analysis

Differentially expressed gene sets for four comparative pairs (up- and downregulated subsets), gene sets for transition curve pattern groups, and gene sets for weighted gene co-expression network analysis modules were used for over-representation analysis using clusterProfiler v3.10.1 package^54^ in R with parameters set as adjusted *p* < 0.05 (Benjamini–Hochberg method^53^) and max gene size = 1000. To understand the biological functionality of gene sets, we considered curated gene sets from the Molecular Signature Database (MSigDB) v7.4 (released in April 2021)^55,56^. Specifically, we used the curated gene sets of KEGG^57^, Reactome^58^, and Wikipathways^59^ for canonical pathways (C2:CP) and biological process, cellular component, and molecular function Gene Ontology terms (C5:GO)^60,61^. We used Gene Ontology biological processes as the main set of functional and pathological annotations for defining disease status in this study. For cell-type enrichment analysis, we used mouse primary somatosensory cortex cell type markers^62^. In addition, we also performed cell-type enrichment with human cell type markers, including embryonic cortex-specific cell type markers^63^, from cell type signature gene sets (C8) in the MSigDB, as well as entorhinal cortex cell type markers from postmortem AD patients^64^.

### Data augmentation and normalisation for training

The original dataset comprised 20 samples from 4 different age–phenotype groups (*n* = 5/group). To increase the size of the training dataset, we performed data augmentation based on the ten pairwise combinations within each group. Under a combination between two original samples ($s1$ and $s2$), we created nine augmented samples (${S}_{aug}$) by linear interpolation ${S}_{aug}=x\times s1+(1-x)\times s2$, where $x= [0.1, \dots , 0.9]$. Hence, 360 augmented samples (4 groups $\times$ 10 pairs $\times$ 9 linear interpolations) were generated, which were combined with the 20 original samples for GAN training.

We applied the standard-distribution-like scaling operations as follows:

1 $$rescaled \; RLD=\frac{SR}{\left(3.918{\upsigma }_{SR}\right)}+0.5,$$

where $SR$ is defined for a sample ($i$), a gene ($j$), and a condition ($k$) as follows:

2 $$SR\left[i,j\right]=\frac{\left(RLD\left[i, j\right]- \mu \left[j\right]\right)}{{\sigma }_{IQR}\left[j\right]},$$

where $RLD\left[i, j\right]$ is the RLD of the $j$ genes over the $i$ samples and $\mu \left[j\right]$ is the mean of the $j$ genes over all samples. In this study, we employed a complex denominator ${\sigma }_{IQR}\left[j\right]$ for each gene. We evaluated ${\sigma }_{GM}[j]$, a geometric mean between $\sigma \left[j\right]$ and $\mathrm{max}(\sigma \left[k,j\right])$, where $\sigma \left[k,j\right]$ is the standard deviation of $j$ genes over all samples and $\sigma \left[k,j\right]$ is the standard deviation of $j$ genes over the samples with a $k$ condition. In a distribution over ${\sigma }_{GM}[j]$, we selected ${\sigma }_{IQR}[j]$ conditionally as follows:

3 $${\sigma }_{IQR}[j] =\left\{\begin{array}{ll}{ \sigma }_{GM}\left[j\right] & \quad if \;\;\; Q1 < { \sigma }_{GM}\left[j\right] < Q3, \\ Q1 & \quad if \;\;\; { \sigma }_{GM}\left[j\right] \le Q1, \\ Q3 & \quad if \;\;\; { \sigma }_{GM}\left[j\right] \ge Q3,\end{array}\right.$$

where $Q1$ is the first quartile (25th percentile) of the distribution over ${\sigma }_{GM}\left[j\right]$ and $Q3$ is the third quartile (75th percentile) of the distribution over ${\sigma }_{GM}\left[j\right]$. Through this complex normalisation procedure, we obtained a similar distribution to the normal distribution, which is critical to generate realistic fake data that simulate the real data in a highly precise manner. The standard-scaled RLDs were rescaled so that 95% of the values fell within the range [0,1], which is appropriate for the leaky ReLU activation function used in our GAN model.

### Network architecture and hyperparameters

We adopted the advanced Wasserstein GAN with a gradient penalty, which consists of two networks, generator and discriminator, and contains several advanced features for learning^65,66^. We implemented the two networks composed of fully connected layers (FC) and a leaky rectified linear unit (ReLU) activation function using TensorFlow v.2.2^67^ with Keras^68^ in Python v.3.7. We set the number of hidden layer units for the generator and discriminator to 450 and 270, respectively. Thus, the architecture of the generator and discriminator were input FC (100)—leaky ReLU—hidden FC (450)—leaky ReLU—hidden FC (450)—leaky ReLU—output FC (3767) and input FC (3767)—leaky ReLU—hidden FC (270)—leaky ReLU—hidden FC (270)—leaky ReLU—output FC (1), respectively (see Supplementary Fig. 2). In addition, we set the initial random weight parameters in the generator within the range [− 0.3, 0.3] and generated random variables in the latent space determined by the distribution of the rescaled regularised logarithmic profile data as previously described^26^. The weights were updated by learning based on the loss of the Wasserstein GAN with gradient penalty (gradient penalty = 10) with the Adam optimiser (learning rate = 1e−5) for 300,000 epochs with a minibatch size of 32. We evaluated the generator output performance at multiple checkpoints using t-distributed stochastic neighbour embedding (t-SNE)^69^ and correlation between real and generated samples. The above hyperparameters were adjusted by trial and errors through our previous work and this work. We also tested the robustness of the results under variations of network architecture with six different pairs of hidden layer units for the generator and the discriminator. We selected an optimized hyperparameters, for which detailed procedures and results were described in our previous work.

### Latent space interpolation

We randomly generated 10,000 latent vectors ($z$) at each epoch and gene expression profile vectors $x=G\left(z\right)$ using the generator $G$. We obtained the top 10 high-correlation profile vectors ${G(z)}_{high}$ for an augmented sample, estimated the average latent space vector $<z>$ over 10 ${G(z)}_{high}$, and generated an averaged profile $G(<z>)$. In this way, we generated 380 realistic fake gene profiles $G(<z>)$ corresponding to the 380 augmented data in each epoch—95 realistic fakes for each condition. A difference vector was calculated by subtracting the averaged latent vectors for each condition as $\Delta ={\sum }_{i}^{95}{z}_{AD6M(i)}/95-{\sum }_{i}^{95}{z}_{WT6M(i)}/95$ for two states, for instance, six-month-old wild type (WT6M) and transgenic AD (AD6M) animals. The simulated transitional states between WT and AD were estimated by the arithmetic equation $z\left(t,i\right)={z}_{WT6M(i)}+t\Delta$ and the generator $G\left(z(t,i)\right),$, where $t=[0, 1]$ and ${z}_{WT6M(i)}$ represent 95 latent vectors for WT6M. We calculated the TCs $T(t)$ for 3767 genes by averaging, where $T(t)={\sum }_{i}^{95}G(z\left(t,i\right))/95$. The above averaging processes using all augmented WT states were necessary due to the high sensitivity of the difference vectors (Δ) and the irregular curve patterns, which sometimes occurred at the starting points (${z}_{WT}$). Finally, we averaged the TCs $<T\left(t\right)>$ over 100 epoch points (225–275 K).

### Clustering and grouping of TC patterns

The extracted TCs were clustered to analyse molecular patterns of disease progression. The four-way TCs of 3767 genes were merged into 56 clusters using affinity propagation clustering (APC)^70^ in the scikit-learn library with default parameters^71^. Since the number of genes in each cluster was too small to perform GOBP enrichment analyses, we grouped the 56 clusters into eight pattern groups (P1 to P8) by direction (upwards or downwards) of patterns of each TC and one undefined pattern group.

### Protein‒protein interaction network

To assess and visualise protein‒protein interactions among grouped genes, we used STRING v.11.5, which provides an integration of such interactions, including both direct physical and indirect functional associations^72^. The resulting data were visualised using Cytoscape^73^.

### Analysis of postmortem human expression profiles

The two preprocessed RNA-seq gene expression datasets of postmortem human AD studies (temporal cortex in Mayo clinical data [syn5550404; control *n* = 78 (Braak stage 0-III) and AD *n* = 82 (Braak stage IV-VI)]^74^ and prefrontal cortex in ROSMAP data [syn3219045; control *n* = 120 with Braak stage 0-III and CERAD score *n* = 61 (No AD), 23 (possible), 28 (probable), 8(definite) and AD *n* = 154 with Braak stage IV-VI and CERAD score *n* = 9(No AD), 1 (possible), 55 (probable), 89 (definite)]^75^ were obtained from the AMP-AD Knowledge Portal, available on Synapse (https://www.synapse.org). Then, the gene expression profiles were used to calculate module eigengenes (MEs, the first principal component of each module). The *p* values were obtained using the unpaired two-sample Wilcoxon rank-sum test.

### Weighted gene co-expression network analysis (WGCNA) and module identification

To identify gene co-expression networks, we utilised the union set of 3767 DEGs as in the GAN training except removing three confounding genes (*Erv3, Rassf2*, and *5330413P13Rik*) from the four comparative pairs by applying WGCNA v.1.70-3 R package^76^ as previously described^10^. A thresholding power of six was chosen (as it was the smallest threshold that resulted in a scale-free R2 fit of 0.8), and the network was created using the function blockwiseModules to calculate the componentwise minimum values for topologic overlap (TOM). The resulting modules were used to calculate module eigengenes (MEs).

### Permutation analysis as a statistical test on a small sample size

Our new GAN-based pipeline to analyse bulk RNA-seq datasets utilises a very small number of samples compared to other deep-learning-based methods using single-cell NGS datasets. We performed a statistical test by permutation analysis to validate our biological findings derived from a small sample size. We permutated samples in three ways: (1) across all 20 samples, (2) within the same phenotypes, and (3) within the same age groups. WGCNA and GAN analyses were repeated twenty times at each permutation condition. We observed loss of module significance in the most WGCNA analyses (Supplementary Fig. 9a) and the original data are well separated from permutated data by visualising module correlations and p values of 21 datasets (Supplementary Figs. 9b and 10). The GAN training could be performed successfully, while the clustering patterns in the t-SNE plots were destroyed (Supplementary Fig. 11), which supports fake samples for estimating TCs can be generated successfully. However slopes of TCs were changed randomly, which were checked by direct visualisation of TCs (Supplementary Fig. 12) and scatter plots of starting and end points of TCs of 12 selected genes belonging to three patterns (Supplementary Fig. 13). Hence, samples in each group are demonstrated to be well characterised with their own features and populations, which supports that biological findings obtained by the GANs analytic pipeline would be acquired based on their phenotypic differences.

## Supplementary Information

Supplementary Figures.

Supplementary Table 1.

Supplementary Table 2.

Supplementary Table 3.

Supplementary Table 4.

## Data Availability

The source code used in this study is openly available in GitHub at https://github.com/KBRI-Neuroinformatics/WGAN-for-toupathy.
