# Supplementary material for: Investigation of early molecular alterations in tauopathy with generative adversarial networks
Source: Sci Rep. 2023 Jan 13;13:732. doi: 10.1038/s41598-023-28081-6 (PMC9839697; doi:10.1038/s41598-023-28081-6)
Supplement: Supplementary file 1 — Supplementary Figures. [file 41598_2023_28081_MOESM1_ESM.docx]

Supplementary Information

**Investigation of early molecular alterations in tauopathy with generative adversarial networks**

Hyerin Kim^1,+^, Yongjin Kim^1,+^, Chung-Yeol Lee^1,+^, Do-Geun Kim^1^ and Mookyung Cheon^1,^*

^1^Dementia Research Group, Korea Brain Research Institute (KBRI), Daegu, 41062, Korea

^+^These authors contributed equally to this work

* email: [mkcheon@kbri.re.kr](mailto:mkcheon@kbri.re.kr)

**Supplementary Table 1**. Gene ontology enrichment analysis results for up- and down-regulated genes of four comparative pairs.

**Supplementary Table 2**. Gene lists belonging to P1 ~ P8 pattern groups

**Supplementary Table 3**. Gene ontology enrichment analysis results based on gene lists of P1~P8 pattern groups

**Supplementary Table 4**. Gene lists of WGCNA modules and gene ontology enrichment analysis results

**Supplementary Figures 1~11**


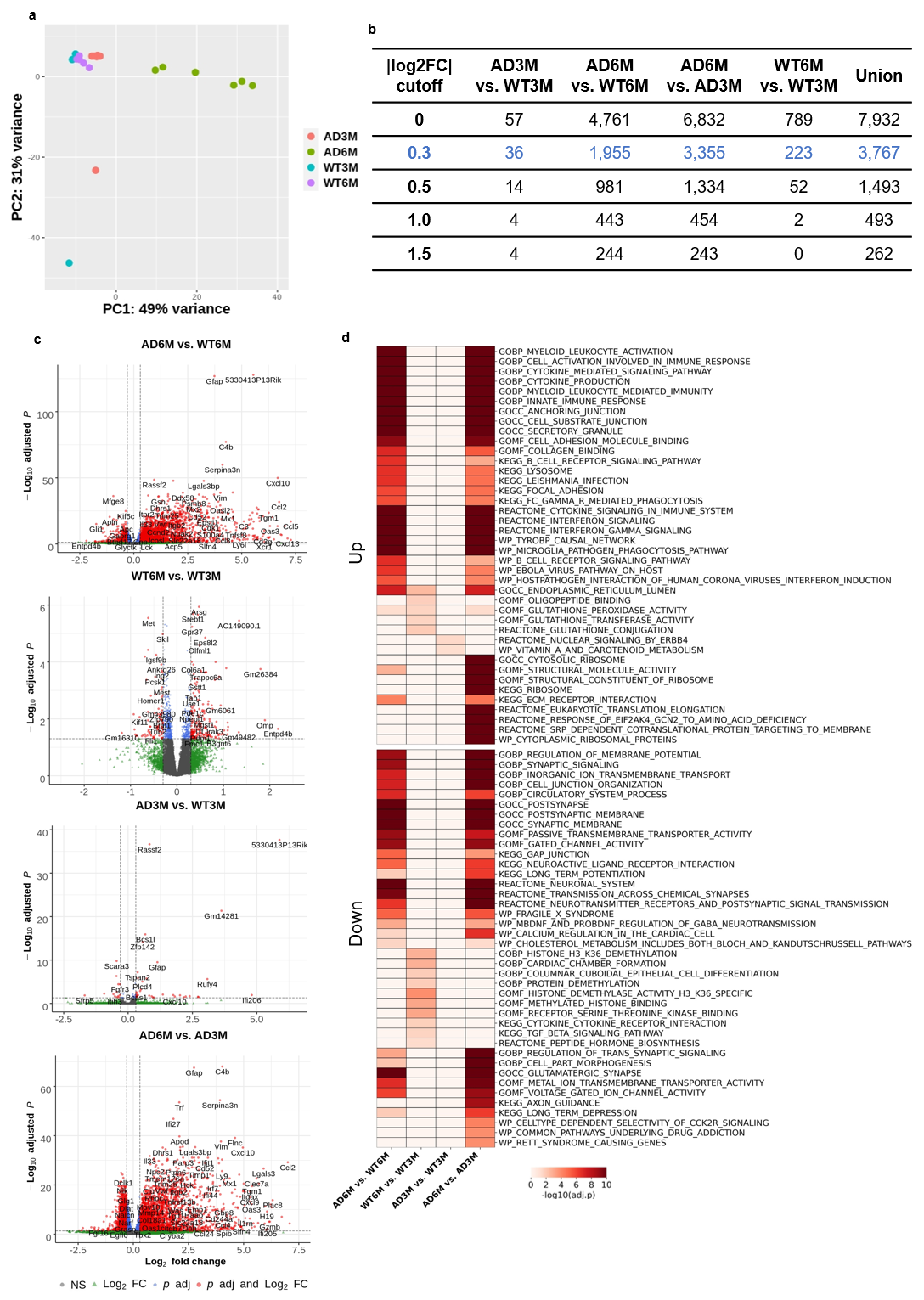


**Supplementary Figure 1. RNA-seq data reprocessing and differential expression gene analysis results**. **(a)** Principal component analysis for original samples. **(b)** Number of DEGs varying log2FC cutoff values. We chose log2FC cutoff as 0.3 (blue colour). **(c)** Volcano plots of four comparative pairs. **(d)** Pathway enrichment for up- and down-regulated genes of each comparative pair. AD, Alzheimer’s disease; adj.p, adjusted *p* value; FC, fold change; M, months; NS, not significant; WT, wild type.


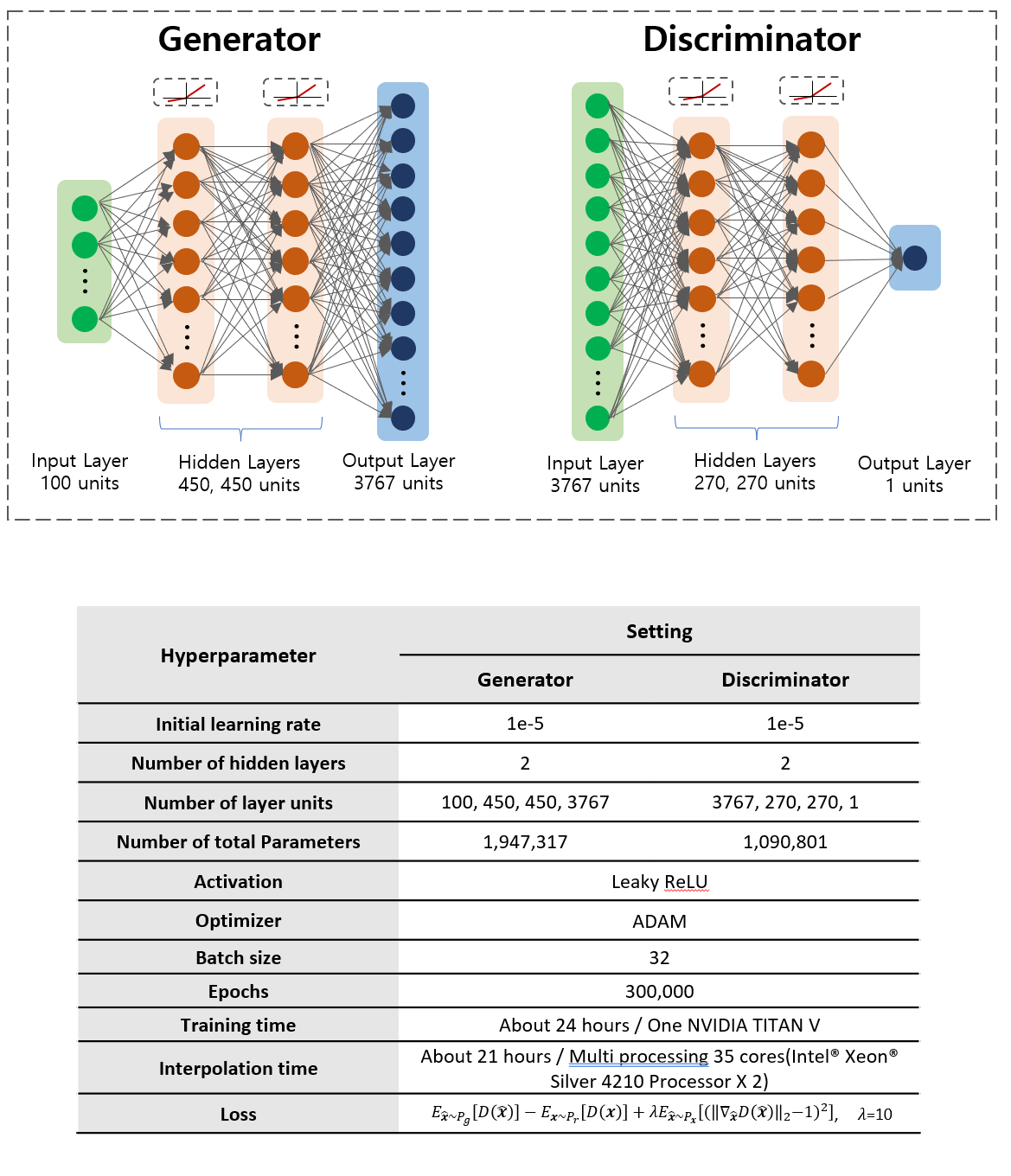


**Supplementary Figure 2.** A figure of network architecture and a table of hyperparameters with experiment environment.


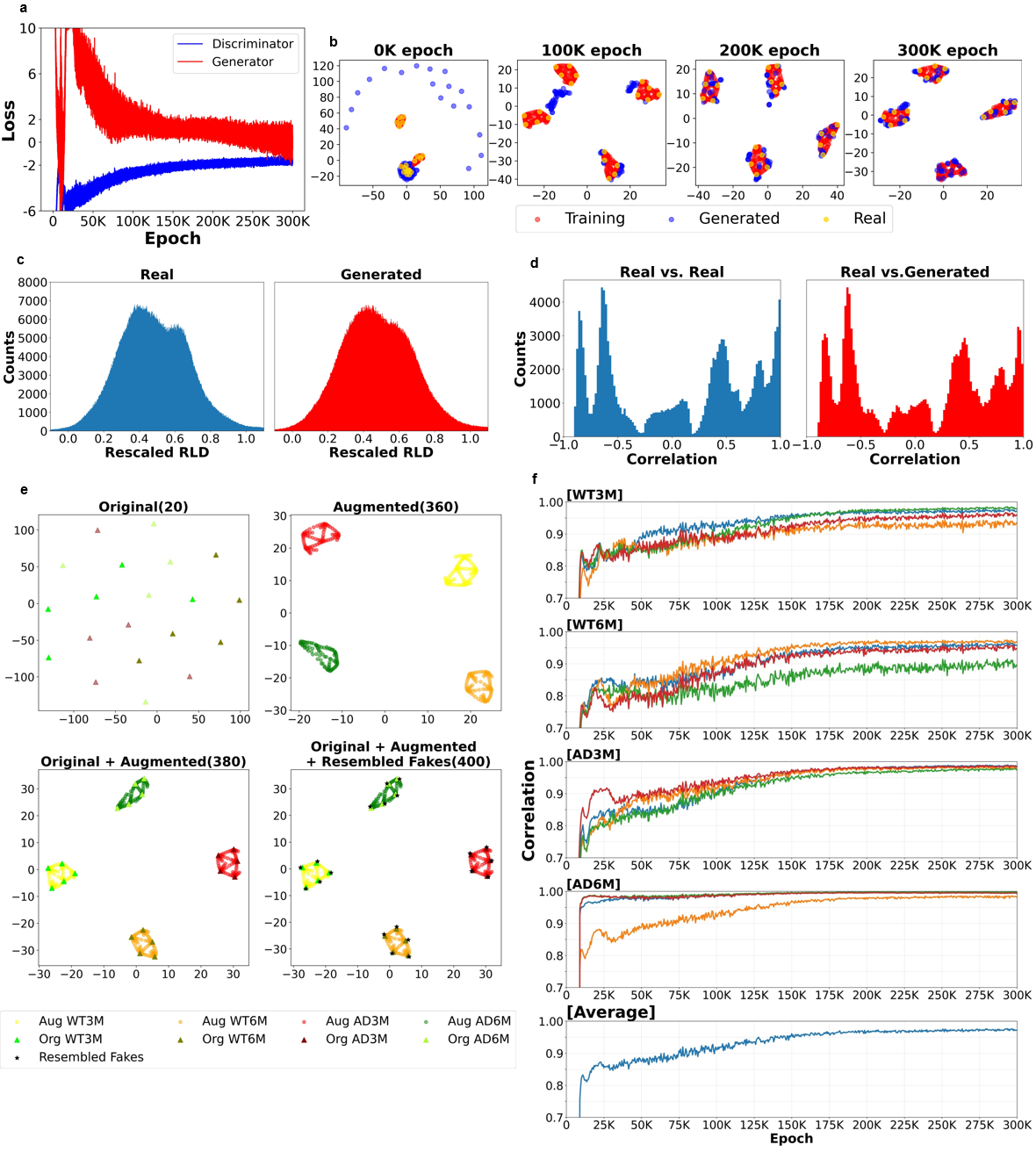


**Supplementary Figure 3. GAN model training and evaluation.** **(a)** Generator and discriminator network loss curves. **(b)** t-SNE projection of 380 training samples, 84 generated samples, and 20 real samples in 0, 100K, 200K, and 300K epochs. **(c)** Distribution of rescaled RLD for real and generated samples after 300K training steps. **(d)** Distribution of correlation coefficients between pairs of real or generated samples after 300K epochs. **(e)** t-SNE projection of 20 real samples (upper left), 360 generated samples (upper right), and 20 resembled generated samples (bottom). **(f)** Correlation coefficient between real and resembled generated samples during training steps. AD, Alzheimer’s disease; Aug, augmented; K, thousand; M, months; Org, original; RLD, regularised-log transformation; t-SNE, t-distributed stochastic neighbour embedding; WT, wild type.


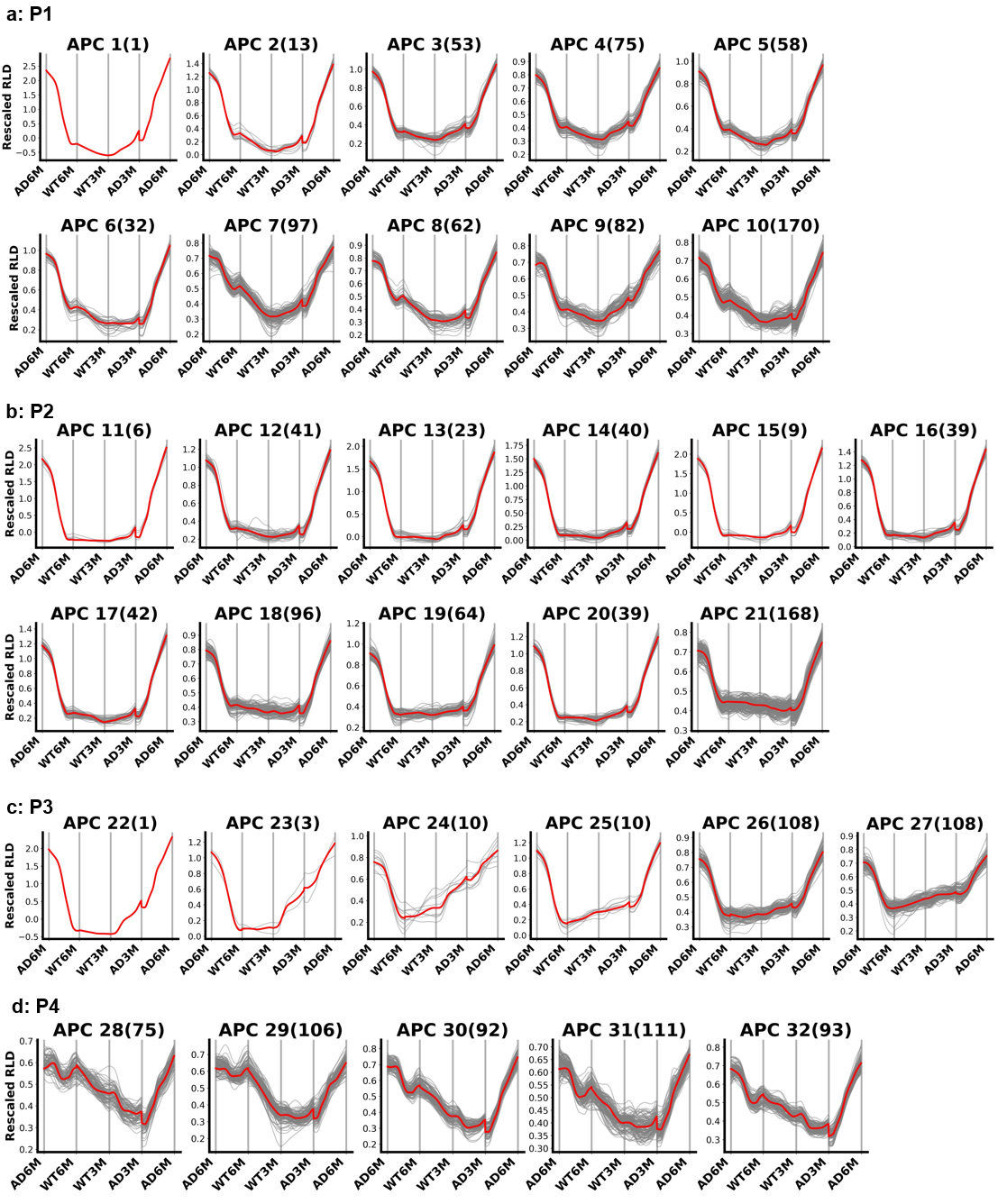


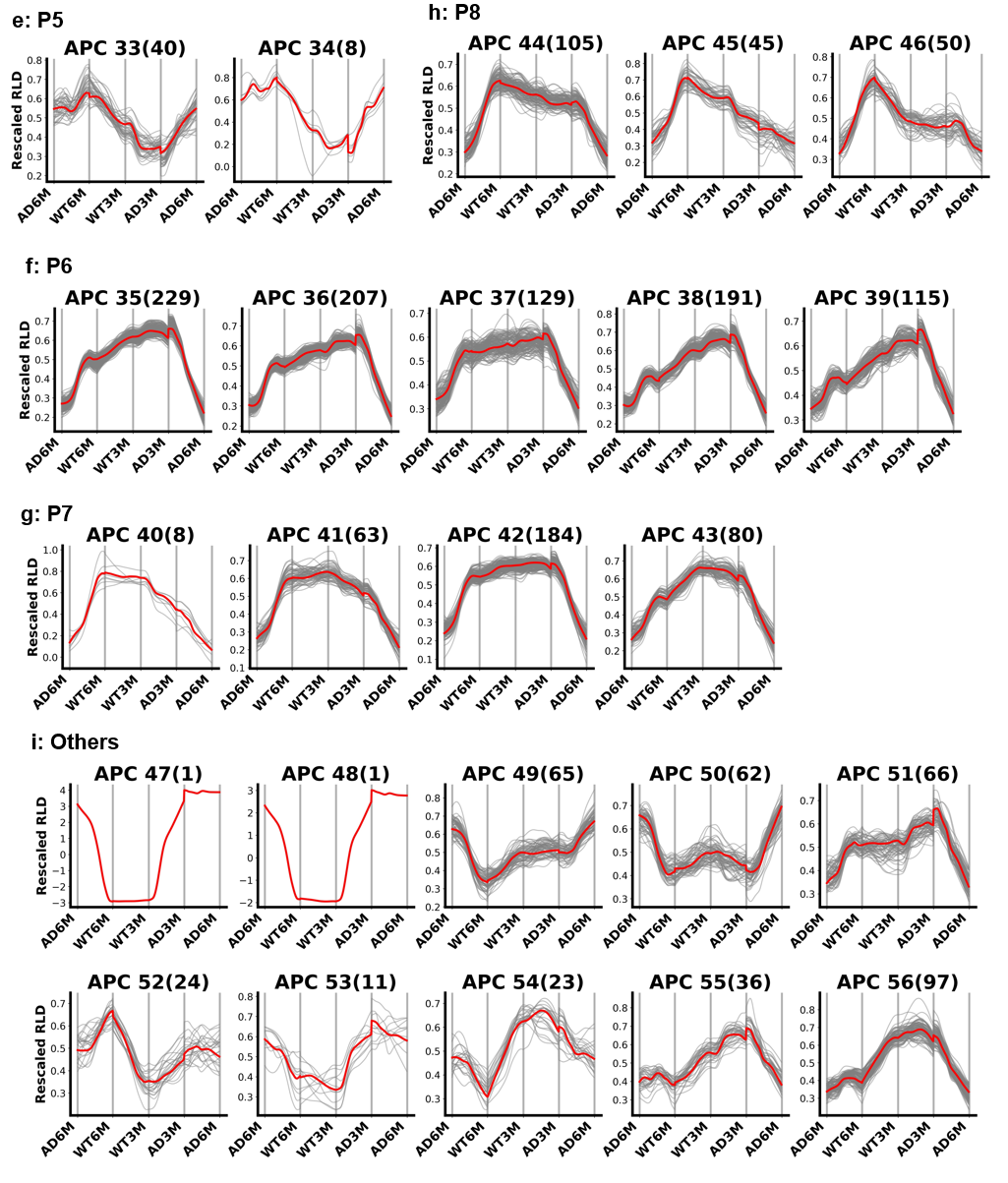


**Supplementary Figure. 4. Pattern clustering and grouping of 3,767 TCs.** (a)-(h) The extracted 3,381 TCs were merged into 46 clusters, which were grouped into eight patterns by direction (upward or downward). i) The 386 ungrouped TCs including two TCs of artificial genes for generating transgenic TPR50 mice (Erv3 and 5330413P13Rik, APC 47 and 48, respectively), the rest of eight outlier clusters could not be merged because they presented undefined patterns. AD, Alzheimer’s disease; APC, affinity propagation clustering; M, months; P, pattern group; WT, wild type.


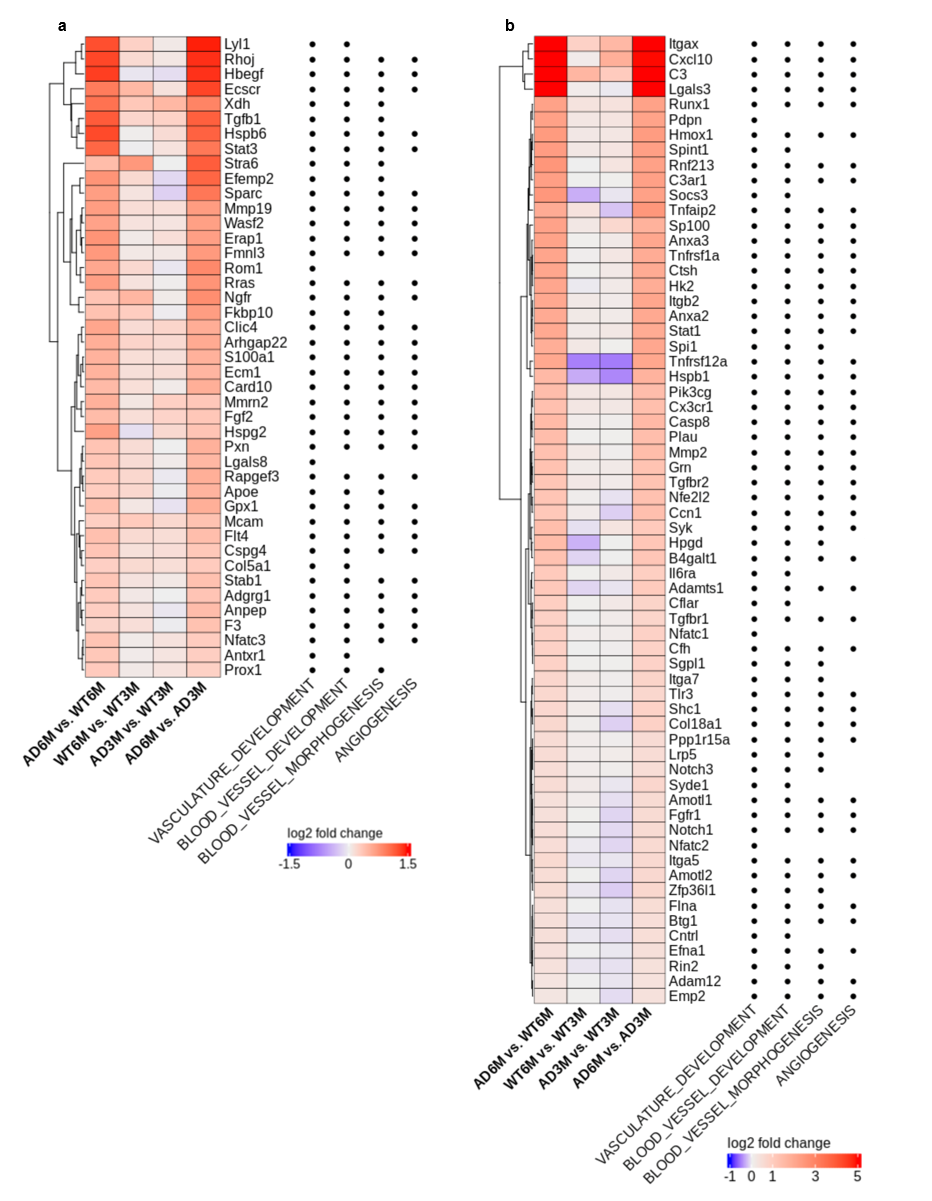


**Supplementary Figure 5. Gene expression heatmap for log2 fold changes in four comparative pairs and associated Gene Ontology pathways.** The numbers of genes were **(a)** 43 in P1 and **(b)** 64 in P2. AD, Alzheimer’s disease; M, months; WT, wild type.


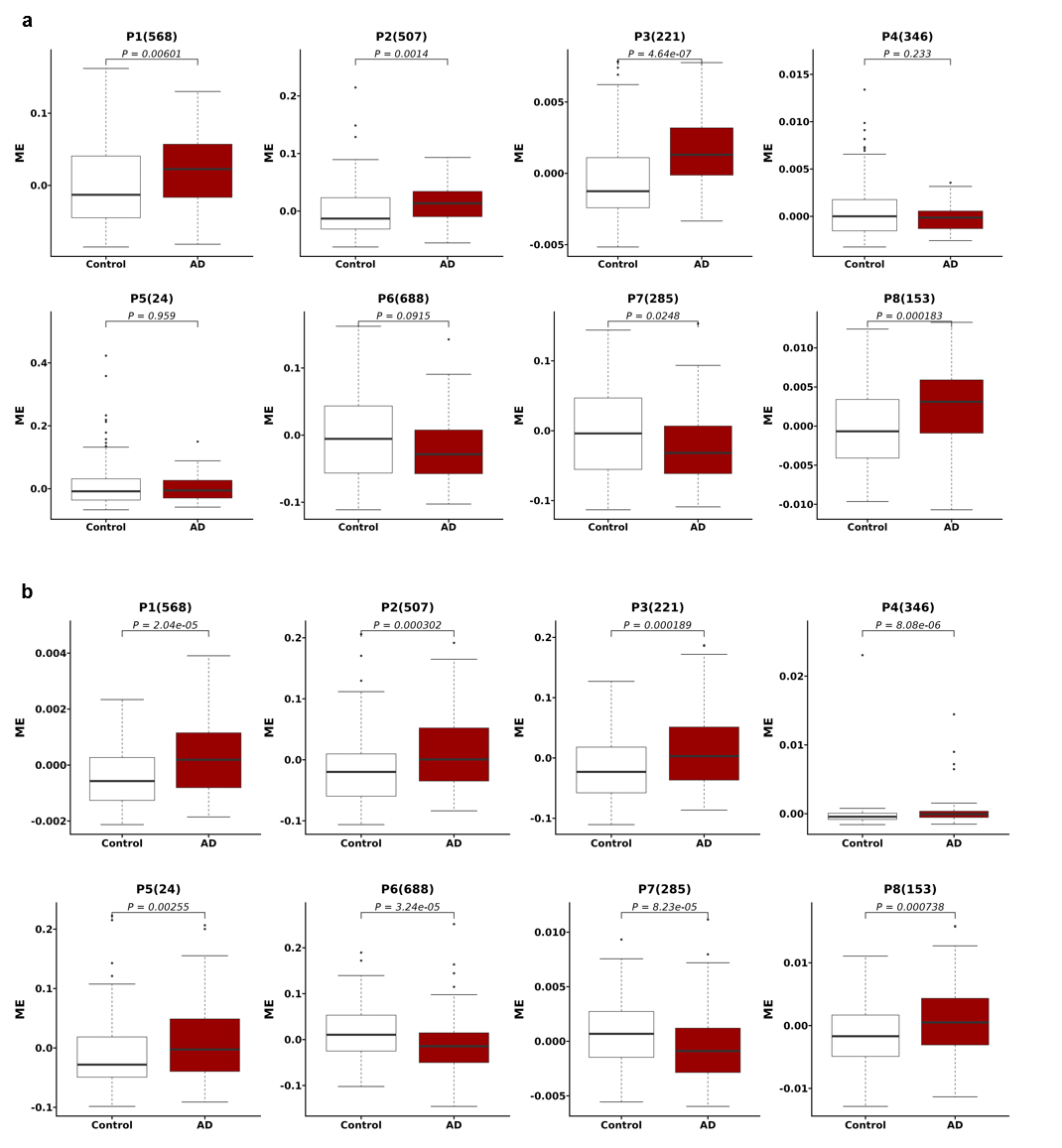


**Supplementary Figure 6. Module eigengenes for all pattern groups in human AD studies. (a)** Temporal cortex data from the Mayo clinic dataset (control *n*=78 with Braak=0-III ; AD *n* = 82 with Braak=IV-VI). **(b)** Prefrontal cortex data from the ROSMAP dataset (control *n* = 120 with Braak=0-III; AD *n* = 154 with Braak=IV-VI). ME, module eigengene; P, pattern group.


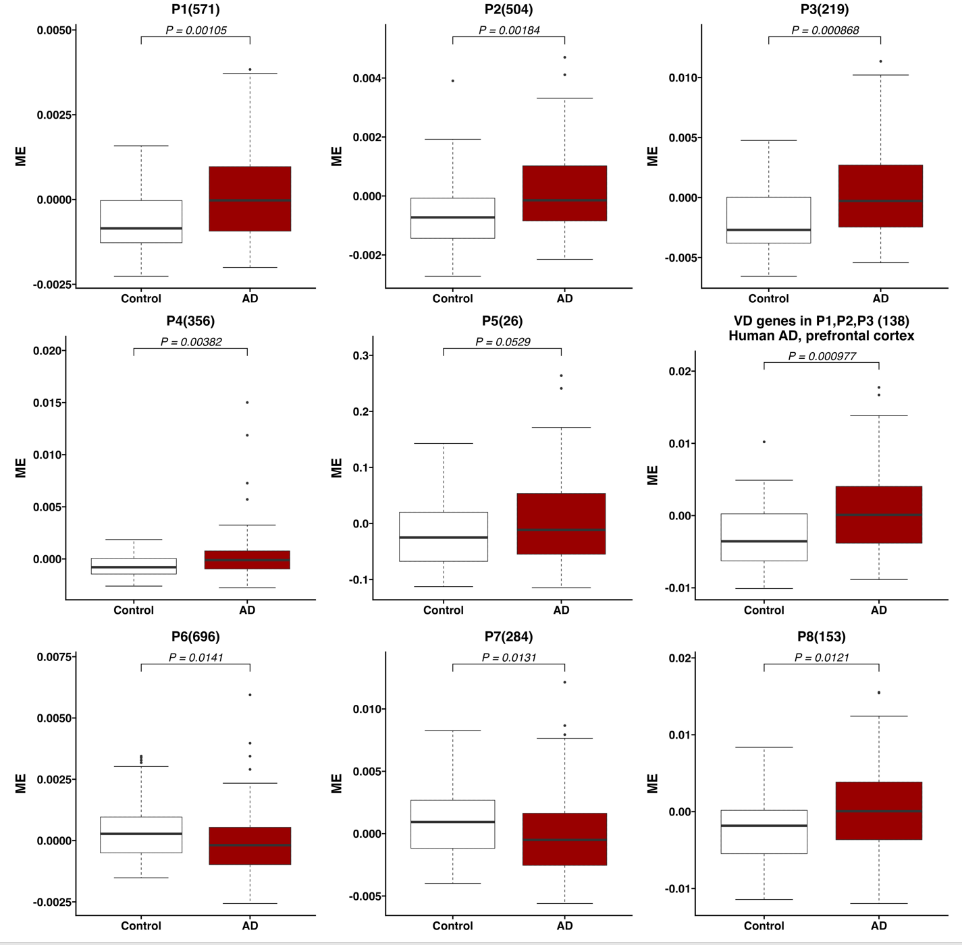


**Supplementary Figure 7. Module eigengenes for all pattern groups in the ROSMAP dataset as a sensitivity analysis.** Prefrontal cortex data from the ROSMAP dataset (control *n* = 37 with Braak=0-I and CERAD score *n*= 24(No AD), 7(possible), 6(probable); AD, *n* = 154 with Braak=IV-VI and CERAD score *n*= 9(No AD), 1(possible), 55(probable), 89(definite)). As a sensitivity analysis, we evaluated module eigengenes with more strict control data. Unfortunately, less significant differences between control and AD were observed in most pattern groups compared to the result of Supplementary Fig. 6, which is probably due to the smaller number of samples (*n*=120 for Braak=0~III; *n*=37 for Braak=0-I)


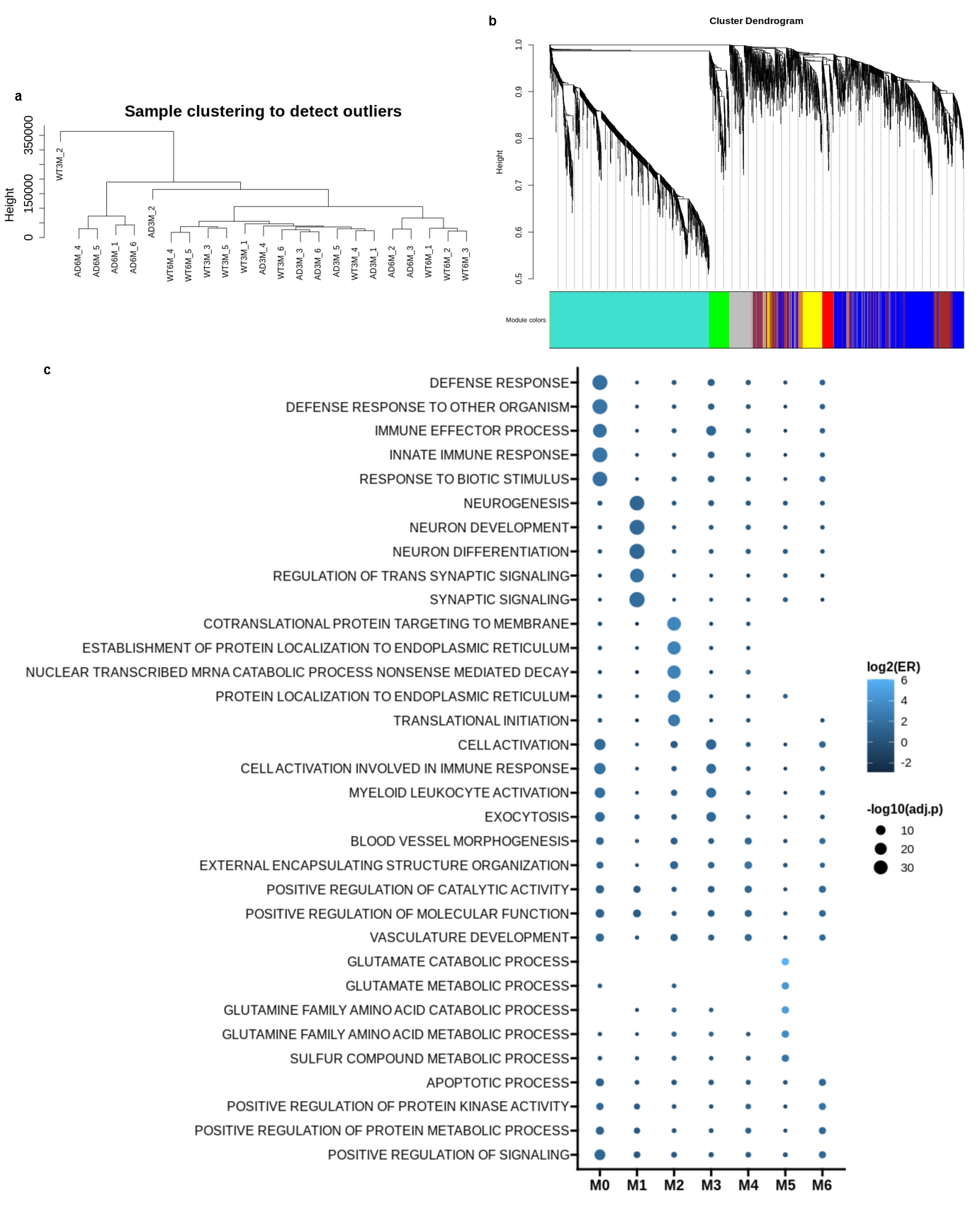


**Supplementary Figure 8. Weighted gene co-expression network analysis. (a)** Hierarchical clustering of the original samples. **(b)** gene dendrogram. Color bar below shows the corresponding modules. **(c)** Gene Ontology pathways of seven WGCNA modules. Statistical significance (adjusted *p* value, dot size) and ER (colour scale) are denoted. Adj.p, adjusted *p* value; ER, enrichment ratio; WGCNA, weighted gene co-expression network analysis.


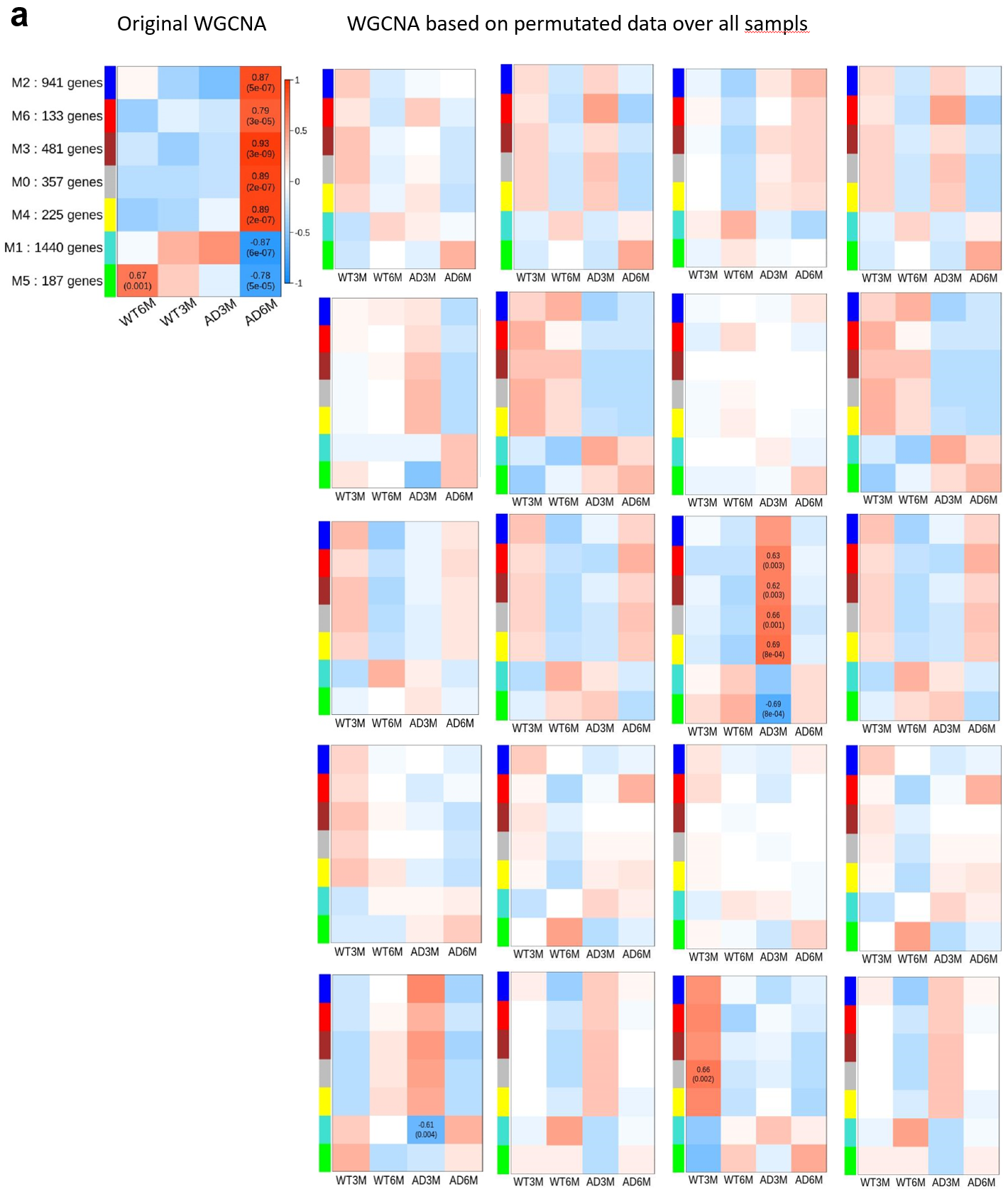

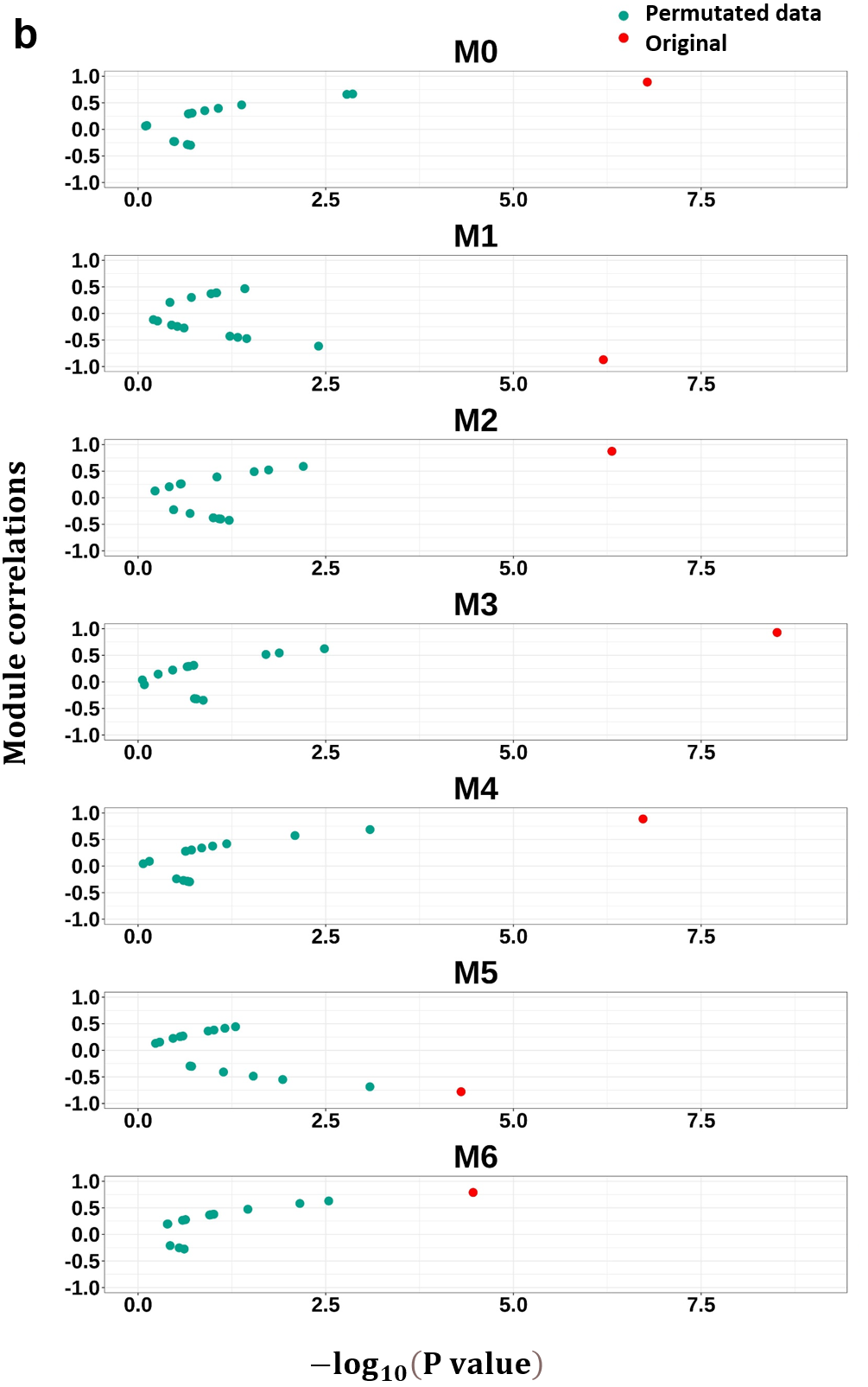


**Supplementary Figure 9. (a)** Heatmaps of module correlations between modules and trait vectors for the original sample dataset and 20 permutated sample datasets. The numbers denoted in the heatmap show correlations and the corresponding p values for associations of module patterns with phenotypes and ages. Loss or weakening of module significance was observed in 19 permutated datasets. For a case, we can observe module significance since four AD6M samples were grouped together by chance. **(b)** Module correlations of original and permutated data, which are chosen as the most significant values among four p-values corresponding to four trait vectors. Module correlations and p-values of original data (red) shows to be well separated from the permutated data.


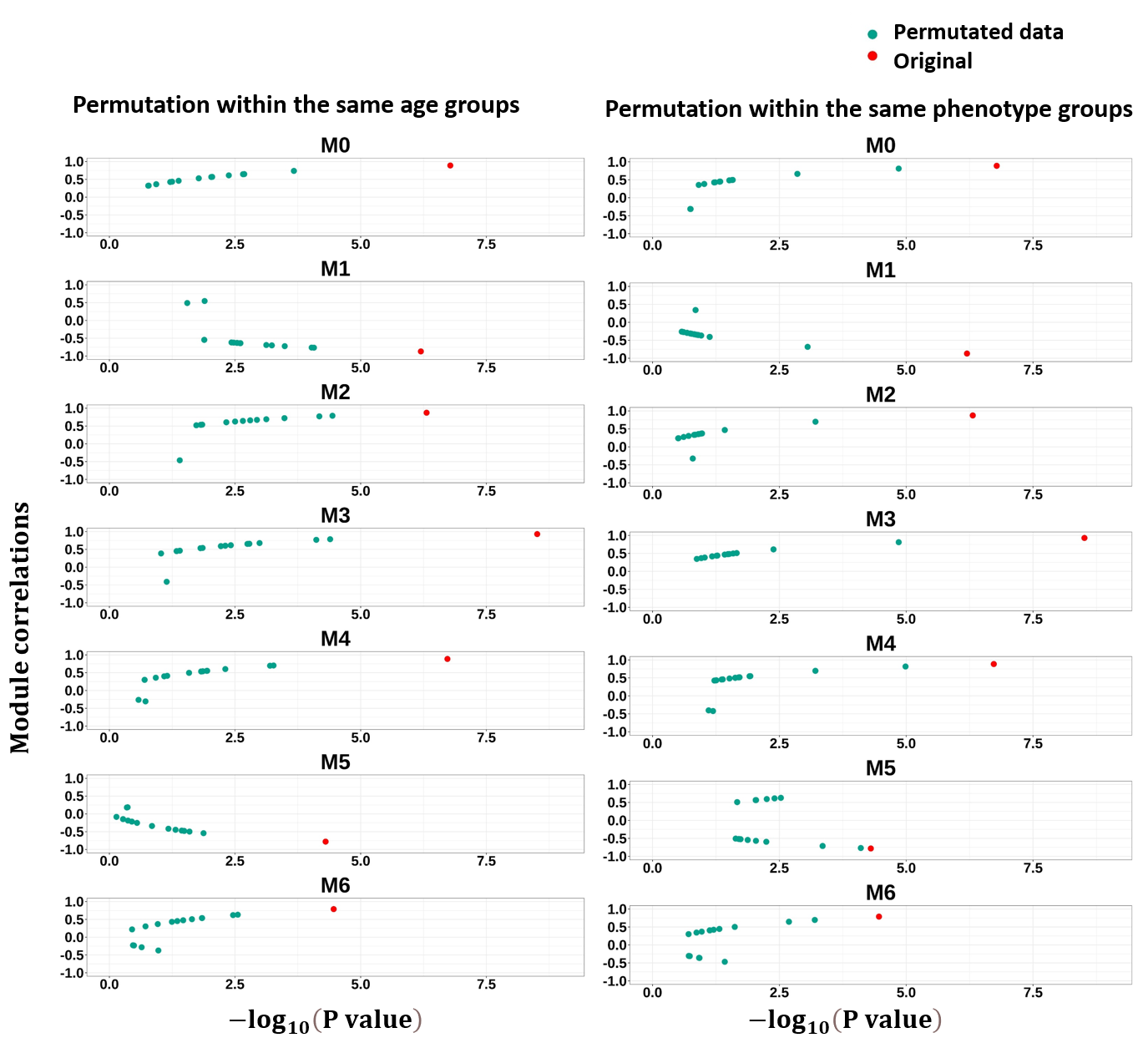


**Supplementary Figure 10. Module correlations of original and permutated sample datasets.** Permutation analyses were performed within the same age groups (left) and the same phenotype groups (right). Although some datasets (M5 module of right figure) are close to the original data in their p values, the original dataset shows the well separated from all other permutated datasets, which supports that the four populations of four groups have clear different distributions depending on age and phenotype differences.


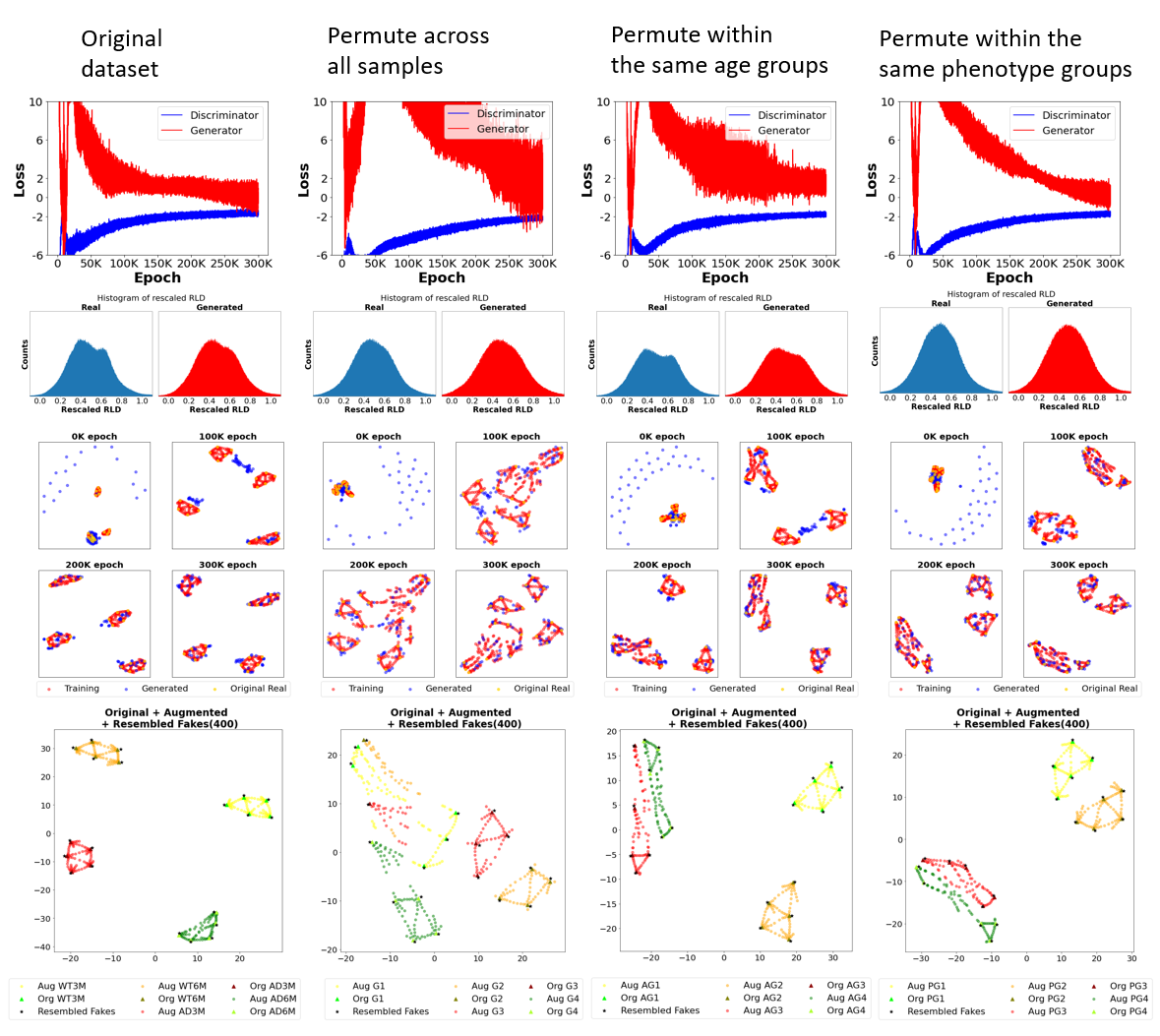


**Supplementary Figure 11. GAN model training and evaluation for the original dataset and permutated sample datasets.** Although we have run GAN trainings twenty times in each way of permutation, we selected a permutated dataset per each condition and presented the training evaluation plots. First panel: Generator and discriminator network loss curves. Second panel: Distribution of rescaled RLD for real and generated samples after 300K epochs. Third panel: t-SNE projection of 380 training samples, 84 generated samples, and 20 real samples in 0, 100K, 200K, and 300K epochs. Fourth panel: t-SNE projection of 20 real samples, 360 generated samples, and 20 resembled generated samples.


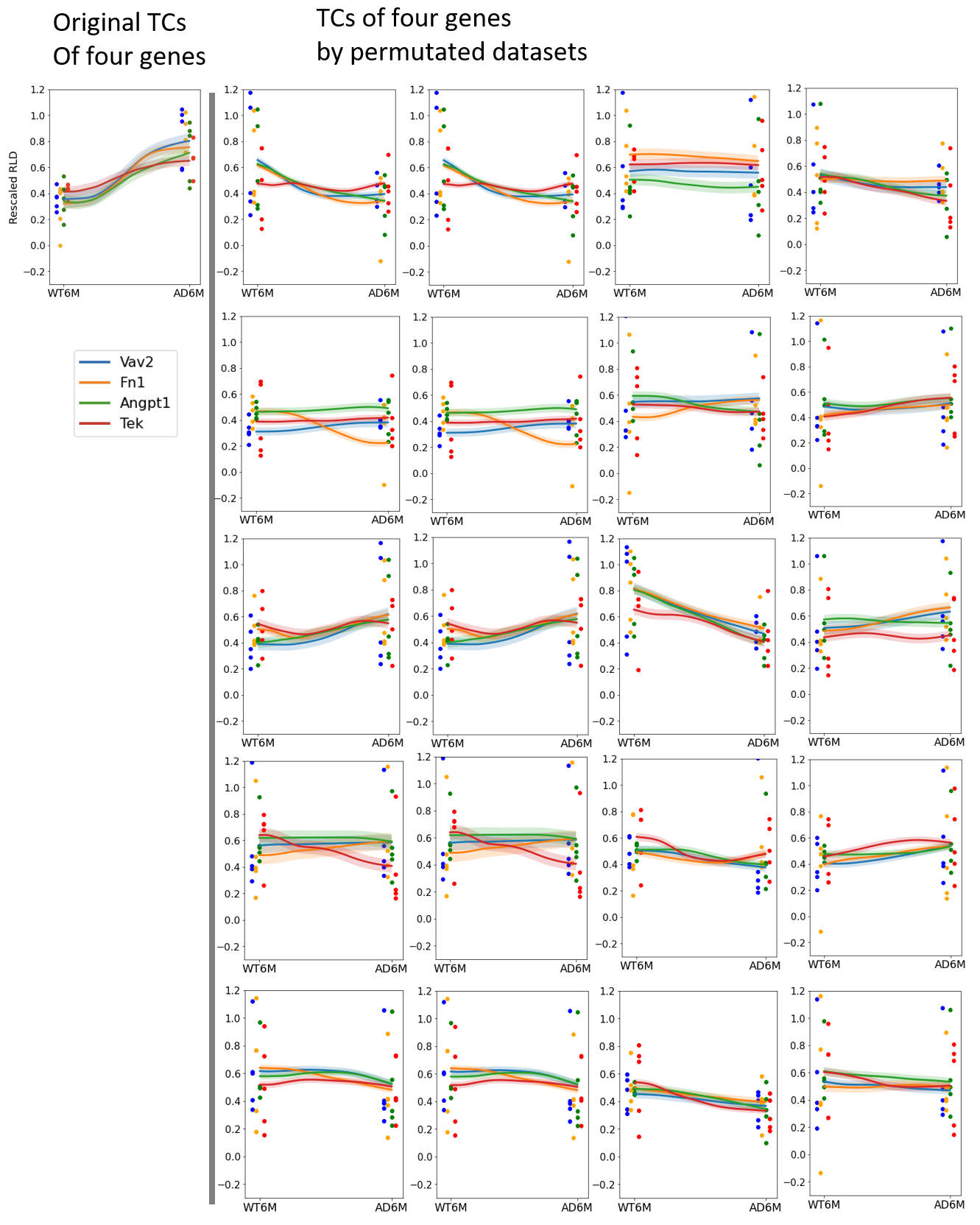


**Supplementary Figure 12. Transition curves(TC1) between WT6M and AD6M for four selected genes in P3 under the original dataset and 20 permuted datasets.** Variations of real sample values (colored dots) in permutated datasets were broaden compared to one of the original dataset. TCs became flat, upward or downward randomly. We presented scatter plots to show visually the random trend of curves. (Supplementary Fig. 13)


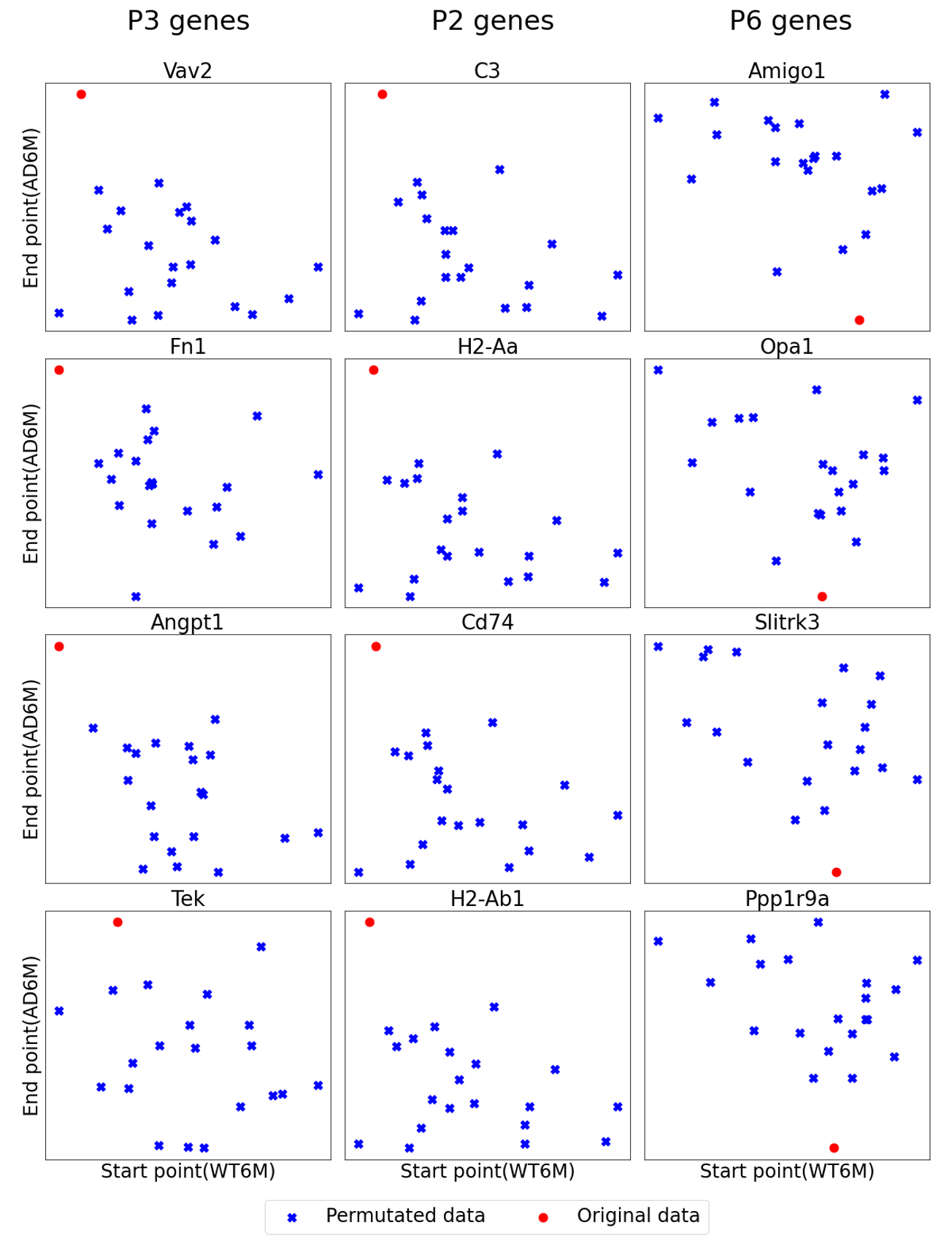


**Supplementary Figure 13. Scatter plots for starting and end points of transition curves (TC1) between WT6M and AD6M.** We selected 12 genes (four P2, four P3 and four P6 genes) to show well separated distribution from 20 permutated data. We confirmed that the starting points(WT6M) of genes do not become maximum or minimum values but the end points (AD6M) become maximum or minimum values, supporting the slopes of all TCs are reduced.
